# *Penicillium* molds impact the transcriptome and evolution of the cheese bacterium *Staphylococcus equorum*

**DOI:** 10.1128/msphere.00047-23

**Published:** 2023-05-23

**Authors:** Ruby Ye, Christopher Tomo, Neal Chan, Benjamin E. Wolfe

**Affiliations:** 1 Department of Biology, Tufts University, Medford, Massachusetts, USA; University of Georgia, Athens, Georgia, USA

**Keywords:** *Penicillium*, *Staphylococcus equorum*, fungal–bacterial interactions, cheese rind microbiome, evolution, RNA sequencing

## Abstract

The observation that *Penicillium* molds can inhibit the growth of *Staphylococcus* was a catalyst for the antibiotic revolution. Considerable attention has been paid to purified *Penicillium* metabolites that inhibit bacteria, but little is known about how *Penicillium* species impact the ecology and evolution of bacteria in multispecies microbial communities. Here, we investigated how four different species of *Penicillium* can impact global transcription and evolution of a widespread *Staphylococcus* species (*S. equorum*) using the cheese rind model microbiome. Through RNA sequencing, we identified a core transcriptional response of *S. equorum* against all five tested *Penicillium* strains, including upregulation of thiamine biosynthesis, fatty acid degradation, and amino acid metabolism as well as downregulation of genes involved in the transport of siderophores. In a 12-week evolution experiment where we co-cultured *S. equorum* with the same *Penicillium* strains, we observed surprisingly few non-synonymous mutations across *S. equorum* populations evolved with the *Penicillium* species. A mutation in a putative DHH family phosphoesterase gene only occurred in populations evolved without *Penicillium* and decreased the fitness of *S. equorum* when co-cultured with an antagonistic *Penicillium* strain. Our results highlight the potential for conserved mechanisms of *Staphylococcus–Penicillium* interactions and demonstrate how fungal biotic environments may constrain the evolution of bacterial species.

**IMPORTANCE:**

Fungi and bacteria are commonly found co-occurring both in natural and synthetic microbiomes, but our understanding of fungal–bacterial interactions is limited to a handful of species. Conserved mechanisms of interactions and evolutionary consequences of fungal–bacterial interactions are largely unknown. Our RNA sequencing and experimental evolution data with *Penicillium* species and the bacterium *S. equorum* demonstrate that divergent fungal species can elicit conserved transcriptional and genomic responses in co-occurring bacteria. *Penicillium* molds are integral to the discovery of novel antibiotics and production of certain foods. By understanding how *Penicillium* species affect bacteria, our work can further efforts to design and manage *Penicillium*-dominated microbial communities in industry and food production.

## INTRODUCTION

Since the first description of *Penicillium* 200 years ago (1), the ubiquity and importance of this diverse group of filamentous fungi have become increasingly apparent. Members of this genus can have both negative and positive impacts on food production as spoilage or beneficial microbes (2, 3). *Penicillium* species are also known for their production of fungal secondary metabolites, including the eponymous antibiotic penicillin, which has saved millions of lives since World War II (4, 5) and sparked both a medical revolution and a subsequent antibiotic resistance crisis (6 - 8).

Despite the importance of *Penicillium* species in the development of antibiotics and other commercially important metabolites, little is known about how these fungi interact with their abiotic and biotic environment in naturally forming microbial communities. Following Alexander Fleming’s famous discovery of *Penicillium* inhibiting *Staphylococcus* growth via penicillin production (4), other studies have screened *Penicillium* species for their ability to inhibit bacteria (9 - 12). But few studies have tested *Penicillium*–bacteria interactions in natural contexts or pairs of microbes that would actually co-occur with one another.

While much of the focus on *Penicillium*–bacterial interactions has been on inhibition of bacteria, *Penicillium* species may also have the ability to stimulate bacterial growth. In cheese production, both yeasts and filamentous fungi can de-acidify cheese rinds, creating a more favorable environment for less acid-tolerant bacteria to colonize and grow, which in turn changes the flavor and quality of the cheese (13, 14). Previous work in our lab and others have also demonstrated bacterial stimulation in the presence of *Penicillium* (15 - 18).

Most studies of fungal–bacterial interactions have only focused on short-term ecological outcomes of these interactions. Longer-term evolutionary consequences of these interactions are unknown. Because of their potential to strongly inhibit or promote the growth of bacteria, *Penicillium* molds could impact the genomic and phenotypic evolution of bacteria through a variety of mechanisms. Bacterial communities could potentially evolve to develop antimicrobial resistance to antibiotics secreted by *Penicillium* in a shared environment. *Penicillium* molds can also provide potential benefits to bacteria that may act as a selective pressure. Previous studies have demonstrated that *Penicillium* species can alter the environment and subsequently impact bacterial fitness, including altering iron availability (15), proteolysis (19), and de-acidification (20). Over time, these changes in resource availability in the abiotic environment could lead to relaxed selection on resource uptake or biosynthesis pathways, which can in turn lead to a permanent dependence on co-occurring fungi for growth (21).

One ideal system to study both short-term and long-term *Penicillium*–bacteria interactions is the cheese rind microbiome. As with many fermented food microbiomes, the cheese rind microbiome is relatively low in complexity, allowing researchers to replicate synthetic communities and elucidate pairwise mechanisms of interactions (15, 16, 18, 22 - 28). A cheese rind typically only contains about five to seven species, which are comparatively easy to isolate and manipulate in a laboratory setting. Cheese rinds, as well as other fermented foods like sourdough and kombucha, are often inoculated with starter cultures that are reused and regularly passaged to recolonize on fresh cheese substrate (26). This standard practice, which strongly resembles a laboratory evolution experiment, allows for ample potential for microbes to evolve and adapt as they interact with novel substrates and each other.

*Penicillium* species are commonly found on cheeses, both as intentionally inoculated starter cultures and environmental contaminants (22, 29 - 31). *Staphylococcus* species have been frequently isolated from cheese rinds where they co-occur with *Penicillium* molds (15, 22, 32). The species *Staphylococcus equorum* is especially common in cheese rinds (15, 22, 32). Previous studies have demonstrated that fungi can promote the growth of *S. equorum* on cheese, possibly by altering iron and free amino acid availability (15), but only one *Penicillium–S. equorum* interaction was assessed. How this bacterium interacts with a range of *Penicillium* species has not been assessed. Additionally, very little is known about how *S. equorum* evolves in the cheese rind environment. While previous studies have demonstrated phenotypic and genomic diversity of *S. equorum* in cheese (15, 27), the evolutionary drivers of this diversity have not been experimentally assessed.

In this study, we paired a short-term comparative RNA-sequencing (RNA-seq) analysis with a co-culture experimental evolution approach to test how *Penicillium* molds can impact both short-term transcriptional responses and long-term evolutionary responses of *S. equorum*. We measured the transcriptomes of *S. equorum* in co-culture with five different *Penicillium* strains spanning four species, predicting that *S. equorum* would shift its transcriptional profile toward tolerance mechanisms to persist with antagonistic *Penicillium* species that produce antibacterial compounds. We next measured the genomic and phenotypic impacts of *Penicillium* on bacterial evolution compared with evolution in monoculture in a 12-week pairwise experimental evolution. We predicted some, but not all, *Penicillium* species would drive *S. equorum* to extinction by secreting antibacterial secondary metabolites. Of the populations in which *S. equorum* persisted, we expected mutations in genes that would confer increased fitness when inoculated with *Penicillium* as well as a loss in fitness when inoculated alone.

## RESULTS AND DISCUSSION

### *Penicillium* species induce broad changes in the *S. equorum* transcriptome

**Fig 1:**
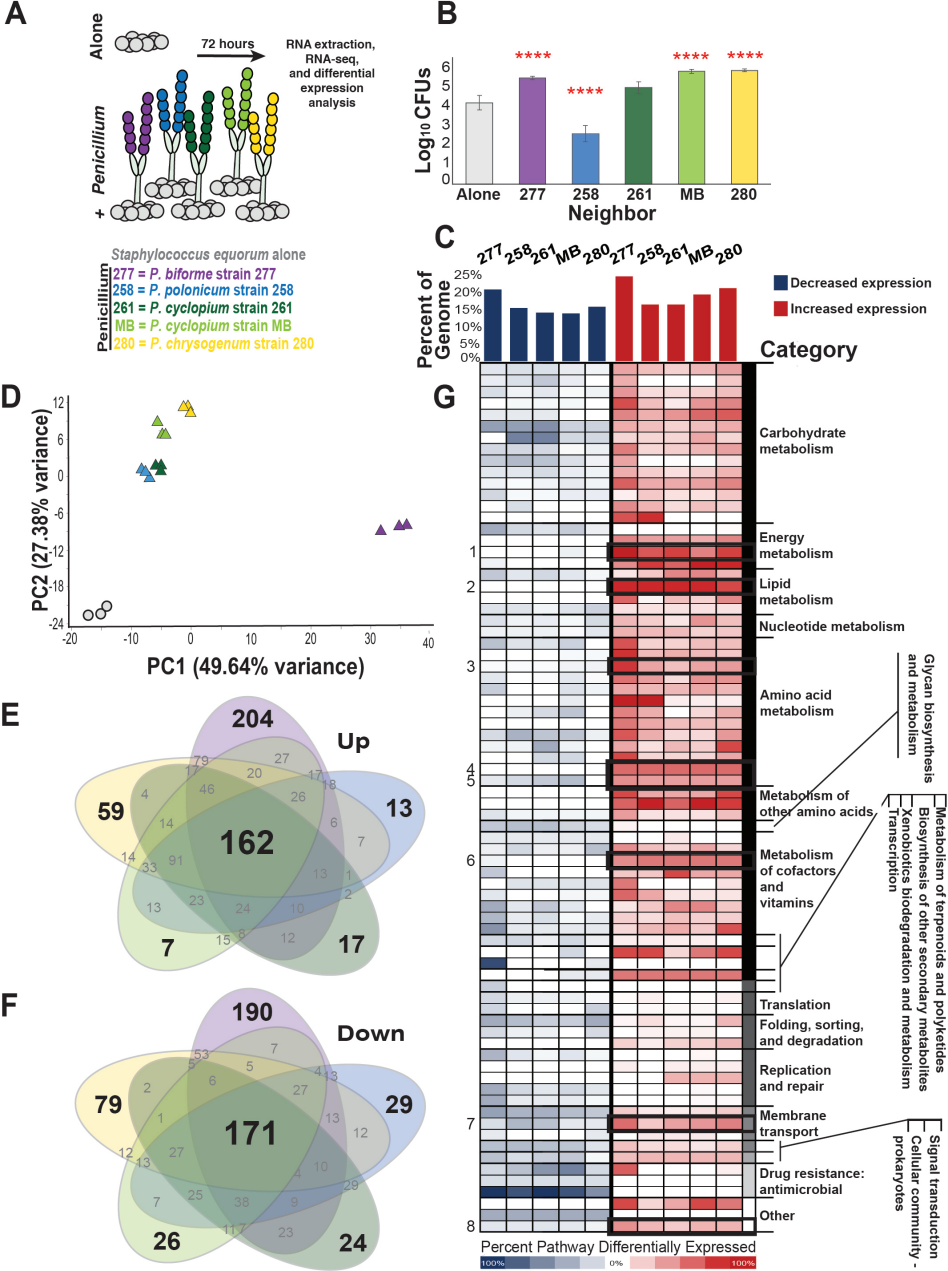
Effects of *Penicillium* species on *S. equorum* mRNA expression in co-culture. Differential mRNA expression in *S. equorum* after 72 hours of growth with one of five *Penicillium* strains compared with growth alone. (**A**) Schematic of the RNA-sequencing experimental setup. (**B**) Log_10_ abundance of colony-forming units of *S. equorum* co-inoculated with a *Penicillium* species compared with growth in monoculture (in gray) (Dunnett’s multiple comparison test, ****P < 0.0001, *n* = 5) (**C**) Percentage of the genes within the *S. equorum* genome that was significantly downregulated (in blue) or upregulated (in red) in the presence of different fungi. Significant differential expression is defined as fold change greater than log_2_ 1 or less than log_2_ −1 when grown with *Penicillium* compared with growth in monoculture. (**D**) Principal component analysis plot of *S. equorum* mRNA expression profiles when co-cultured with *Penicillium* (triangles) or alone (circles); color indicates the *Penicillium* strain. (**E and F**) Number of shared genes significantly upregulated (**E**) or downregulated (**F**) when co-cultured with *Penicillium* (*n* = 3, *P* < 0.05) with the color of the ellipse indicating the *Penicillium* strain. (**G**) Percentage of genes within a KEGG functional pathway significantly downregulated (in blue) or upregulated (in red). Increasing cell color intensity corresponds with increasing percentage of genes within the pathway that was differentially expressed. Bolded boxes highlight functional pathways differentially expressed in all five *Penicillium* treatments, with numbers left of heat map indicating nitrogen metabolism (1), fatty acid degradation (2), glycine, serine, and threonine metabolism (3), tryptophan metabolism (4), phenylalanine, tyrosine, and tryptophan biosynthesis (5), thiamine metabolism (6), phosphotransferase system (7), and metabolism of antibiotics (8). See Table S1 for a full overview of expression levels of all *S. equorum* genes.

To identify putative mechanisms of interactions between *Penicillium* species and *S. equorum*, we measured the transcriptomic response of a previously characterized *S. equorum* cheese isolate, strain BC9 (15). We cultured this bacterium for 3 days on cheese curd by itself or in a mixed co-culture with one of five *Penicillium* strains isolated from natural rind cheeses produced in USA: *Penicillium biforme* strain 277, *P. polonicum* strain 258, *P. cyclopium* strain 261, *P. cyclopium* strain MB, and *P. chrysogenum* strain 280 (Fig. 1A). These *Penicillium* strains span the different taxonomic groups within the genus and have varying impacts on *S. equorum* when grown in co-cultures (Fig. 1B). Differential expression analysis was performed on RNA-seq libraries of *S. equorum* grown alone on cheese curd agar (CCA) and in co-culture with the *Penicillium* strains on CCA.

*S. equorum* exhibited strong transcriptomic responses when co-cultured with a *Penicillium* species regardless of strain identity, differentially expressing from 31% (*P. cyclopium* strain 261) to 46% of the genome (*P. biforme* strain 277), or 851 to 1,270 out of 2,788 predicted genes in the total *S. equorum* genome (Fig. 1C; Table S1). Across all *Penicillium* conditions tested, more *S. equorum* genes were upregulated than downregulated (Fig. 1C), with an average of 365 significantly upregulated and 233 downregulated genes across our five fungal strains (for a threshold of > 5-fold differential expression and corrected *P*-value < 0.05). For comparison, a previous RNA-seq study of *S. equorum* from our lab with similar inoculation densities, an identical medium, and similar incubation times identified 36 significantly upregulated genes and 88 significantly downregulated genes in the presence of *P. solitum* compared with growth alone, as well as 43 significantly upregulated and 137 significantly downregulated when grown with a strain of the fungus *Scopulariopsis* compared with growth alone (15). A study of *S. aureus–Candida albicans* catheter biofilms also observed a much more muted transcriptional response of a bacterium to a fungus with 43 genes with higher expression and 91 with lower expression when *S. aureus* was grown with *C. albicans* compared with growth alone (33). The much higher transcriptional responses we observed compared with past studies are potentially due to the particular biotic environments created by the *Penicillium* strains used in our study.

*S. equorum* responded very differently to *P. biforme* strain 277 compared with the other four *Pencillium* strains (Fig. 1D). Even with this divergent response, we were still able to identify a conserved transcriptomic response of *S. equorum* to all *Penicillium* strains, including 162 upregulated genes (Fig. 1E) and 171 downregulated genes (Fig. 1F). To systematically characterize this core response, we performed pathway enrichment analysis (34) on these 333 genes and identified eight Kyoto Encyclopedia of Genes and Genomes (KEGG) pathways that were upregulated in all five *Penicillium* treatments (Fig. 1G). These eight pathways include nitrogen metabolism, primarily nitrate assimilation (46% of genes within the pathway upregulated), fatty acid degradation (67% of pathway genes upregulated), glycine, serine, and threonine metabolism (21% of pathway genes upregulated), tryptophan metabolism (36% of pathway genes upregulated), phenylalanine, tyrosine, and tryptophan biosynthesis (32% pathway genes upregulated), thiamine metabolism (36% of pathway genes upregulated), phosphotransferase system, primarily components required for galactitol transport (21% of pathway genes upregulated), and metabolism of antibiotics (14% of genes within the pathway). In the KEGG database, nitrogen metabolism includes the following processes: nitrogen fixation, assimilatory nitrate reduction, dissimilatory nitrate reduction, denitrification, nitrification, and complete nitrification. The same gene can be listed under multiple pathways; hence, the full pathway includes genes also involved in the biosynthesis of glutamine, glutamate, and arginine. Similarly, of the 25 genes categorized under metabolism of antibiotics, six genes are involved in glutamate synthesis, six genes in tryptophan synthesis, and three genes in glycine cleavage, further implicating amino acid metabolism as a main response. There were no significantly enriched pathways among the 171 downregulated genes, although we did observe downregulation of at least four genes categorized under cationic antimicrobial peptide resistance in all five treatments (Fig. 1G). However, this was not significant after adjusting for false discovery rate using the Benjamini–Hochberg procedure (*P* = 0.12).

Notably, key genes involved in L-tryptophan biosynthesis and thiamine salvage pathways were upregulated in all *Penicillium* treatments (Fig. S1A and 1B). In addition, staphyloferrin B, an iron siderophore, was also downregulated, although only co-culturing *S. equorum* with *P. biforme* strain 277 and *P. chrysogenum* strain 280 resulted in downregulation of the entire operon, while co-culture with the other three strains (*P. polonicum* strain 258 and both *P. cyclopium* strain 261 and *P. cyclopium* strain MB) only downregulated staphyloferrin B transport genes (SirA-C) (Fig. S1C). We predict that these pathways were upregulated in response to all five *Penicillium* strains in our study due to similarities in how the *Penicillium* metabolized and consumed the media substrate, cheese curd. Specifically, we speculate that the five *Penicillium* species are likely consuming and breaking down complex nutrients in the medium, primarily through the degradation of proteins and complex peptides, breaking down lactose and galactose into derivatives such as galactitol (35), altering fatty acid content (36, 37), and increasing iron availability (18, 38, 39).

The upregulation of thiamine is striking as several other bacterial*–*fungal interaction studies have observed the same response. A previous study in our lab found upregulation of thiamine metabolism in the same *S. equorum* strain against *Scopulariopsis* and *P. solitum* (15). Thiamine has also been implicated in several other bacterial*–*fungal systems, including *Pseudomonas fluorescens* with ectomycorrhizal fungus *Laccaria bicolor* (40), as well as *Bacillus subtilis* with the yeast *Debaryomyces vanriji* (41 ) and *Aspergillus nidulans* (42), all of which found bacterially produced thiamine promoted fungal growth. As such, it is possible that *S. equorum* is similarly upregulating thiamine biosynthesis to promote *Penicillium* growth, potentially as a trade-off for downregulating other metabolic pathways, such as staphyloferrin B transport and synthesis (Fig. S1C). Alternatively, thiamine has been shown to increase sporulation and/or mycelial growth in several *Penicillium* species independently of bacterial interaction (43). As such, it is also possible that *Penicillium* is outcompeting *S. equorum* for exogeneous thiamine, and thus *S. equorum* must upregulate thiamine biosynthesis for its own use. We currently do not have experimental evidence to support any of these possibilities, and future studies are needed to identify how thiamine mediates *Staphylococcus–*fungal interactions.

Strikingly, growth with *P. biforme* strain 277, which resulted in the strongest transcriptomic response from *S. equorum* (Fig. 1C), was also the most unique (Fig. 1D). *S. equorum* expression profiles when grown with *P. polonicum* strain 258*, P. chrysogenum* strain 280*,* and both *P. cyclopium* strains 261 and MB all clustered closely in a principal component analysis of expression profiles, while growth with *P. biforme* strain 277 did not (Fig. 1D). On the gene level, 33% of all differentially expressed genes (DEGs) in the *P. biforme* strain 277 treatment (204 upregulated and 190 downregulated) was unique to that treatment (Fig. 1E and F). Two operons were upregulated, encoding for threonine synthesis and oligopeptide permease (Opp) proteins OppA-F (Fig. S1D).

Opp systems allow bacteria to transport 2–18 amino acid long peptides from the environment into the cytoplasm (44). In environments where free amino acids are low, Opp confers bacteria an important ability to uptake peptides as alternate sources of amino acids and nitrogen (45). *Penicillium* proteases are known to break down casein into oligopeptides in cheese (46, 47). As such, we speculate higher expression of Opp genes when grown with *P. biforme* strain 277 is due to a surplus of oligopeptides in the environment from fungal protease activity that favors oligopeptide uptake. However, the Opp system is also utilized in diverse biological functions, such as quorum sensing, signaling, virulence, and defense, all through the Opp system’s ability to bind extracellular oligopeptides and transport them across the cell membrane (48). Therefore, upregulation of the Opp operon in *S. equorum* could be related to other processes beyond oligopeptide uptake.

**Fig 2:**
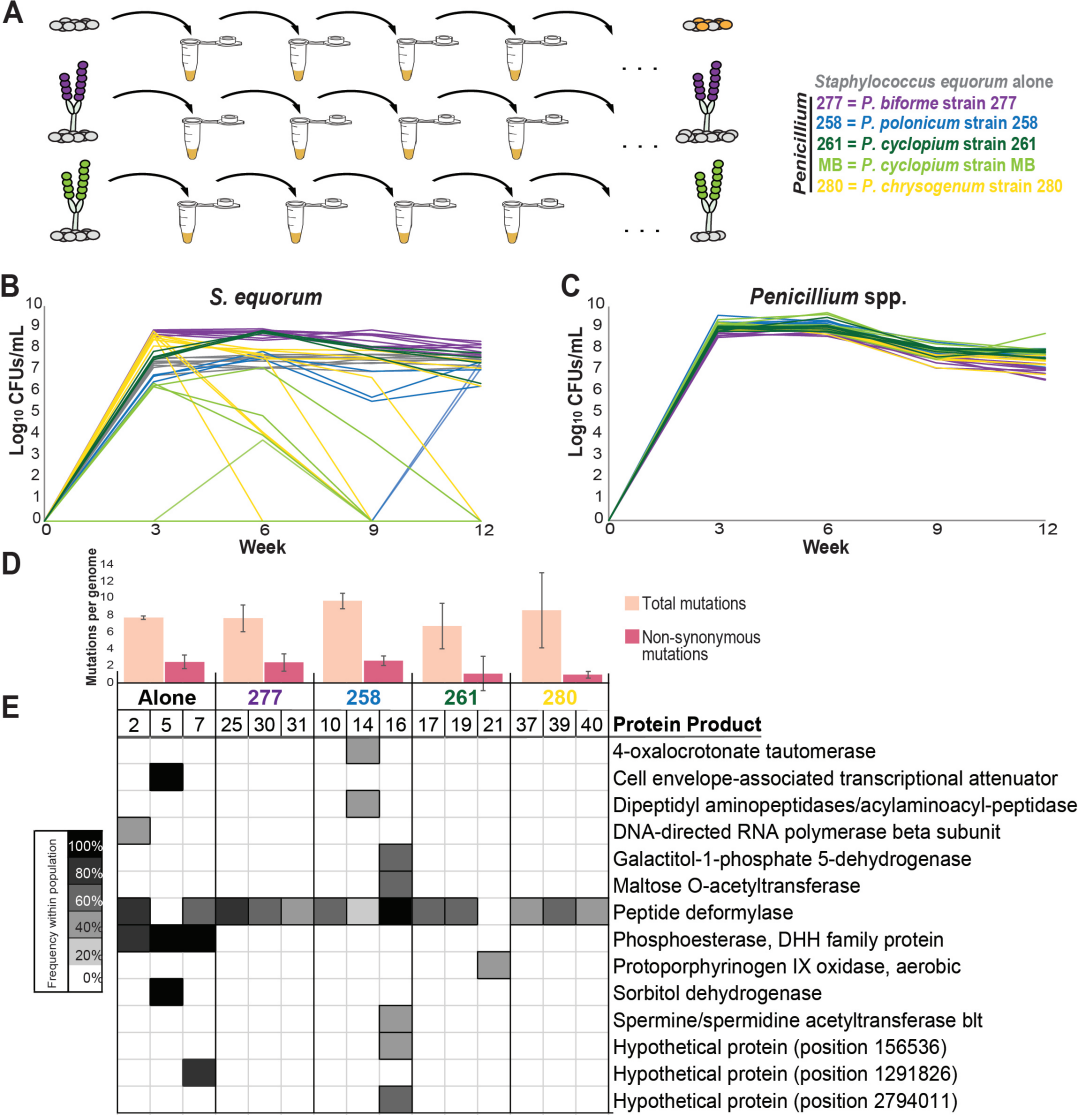
Impact of different *Penicillium* isolates on *S. equorum* evolution. (**A**) Schematic of the experimental evolution setup. Only two of the five fungi are shown for simplicity. (**B**) Abundance of *S. equorum* over the course of the experiment, with each line representing a replicate population of *S. equorum* and colors indicating the *Penicillium* species as described in Fig. 1A. Control (*S. equorum* alone) populations are in gray. (**C**) Abundance of *Penicillium* over the course of the experiment, with each line representing an independent population and colors indicating the *Penicillium* species as described in Fig. 1A. (**D**) Average mutations per genome categorized by treatment, with light pink bars representing total mutations and dark pink bars representing non-synonymous mutations only. (**E**) Genes with mutations in multiple sequenced colonies, with cell color intensity representing percent of the replicate population with a mutated gene. Numbers above the columns (2, 5, 7, etc.) represent the population IDs where colonies were sequenced. See Table S3 for a full overview of all mutations detected.

Growth with *P. chrysogenum* strain 280 also resulted in a divergent transcriptional response, with the transcriptome containing 18% DEGs unique to that treatment alone, compared with 6%–7% of the remaining three strains (*P. polonicum* strain 258, both *P. cyclopium* strains 261 and MB) (Fig. 1E and F). Pathway analysis identified coproporphyrin biosynthesis (Fig. S1E), which has been previously shown to be upregulated in *Glutamicibacter arilaitensis* when grown with *Penicillium* (28 ), to be a unique response of S. equorum to P. chrysogenum strain 280 . In *G. arilaitensis*, it was speculated that the secretion of zinc-chelating coproporphyrin would inhibit *Penicillium* growth (28 ); however, our *P. chrysogenum* strain 280 grows comparable with the other four *Penicillium* strains in our study (Fig. 2C). Furthermore, growth with a more closely related *Penicillium* to the one used with *G. arilaitensis, P. biforme* strain 277, did not induce a similar upregulation in expression in our study, which could possibly be attributed to differences between the two distantly related bacterial phyla.

We also tested whether *S. equorum*’s transcriptomic response to *Penicillium* was correlated with bacterial abundance in co-culture (Fig. S2). At the time of harvest (after 72 hours; Fig. S2A), the number of DEGs was not correlated with *S. equorum* abundance. Interestingly, using long-term *S. equorum* growth data from experimental evolution (see below), we did observe a positive relationship between the number of DEGs and *S. equorum* abundance (Fig. S2B and C). *Penicillium* strains that elicited the strongest transcriptomic response from *S. equorum* tended to also be strains that altered bacterial abundance the most. However, further research co-culturing *S. equorum* with more *Penicillium* and non-*Penicillium species* would be needed to definitively determine whether this trend is significant across the genus and beyond.

### *S. equorum* undergoes limited genomic evolution in co-culture with *Penicillium*

To understand how *Penicillium* species can impact the evolution of bacteria on cheese over a longer period of time, we evolved our *S. equorum* strain alone or with one of five *Penicillium* strains (Fig. 2A). The bacterium was passaged on CCA each week for 12 weeks, with eight biological replicates per treatment. Every third week, we assessed the abundance of both *S. equorum* and the fungi (Fig. 2B and C; Table S2). Given that *S. equorum*’s transcriptomic response was largely conserved across the five *Penicillium* strains (Fig. 1), we predicted similar abundance trends across all five treatments. However, this was not the case (Fig. 2B and C).

After the first 3 weeks, 14 out of 48 replicate communities had non-detectable levels of *S. equorum*, including populations evolved with *P. polonicum* strain 258 (five of eight replicates), *P. cyclopium* strain 261 (four of eight replicates), and *P. cyclopium* strain MB (five of eight replicates) (Fig. 2B; Table S2). By week 12, the remaining *S. equorum* communities grown with *P. cyclopium* strain MB dropped below detectable levels, as well as five communities grown with *P. chrysogenum* strain 280 (Fig. 2B; Table S2). Surprisingly, two communities grown with *P. polonicum* strain 258 contained undetectable levels of bacteria for the first 9 weeks but rebounded on the final plating on week 12. Only when grown alone or with *P. biforme* strain 277 did all eight replicate communities contain detectable amounts of *S. equorum* following the 12-week period (Fig. 2B; Table S2).

*S. equorum* abundance was significantly different depending on the species of *Penicillium* within the co-culture as compared with the control (ANOVA, *F*_5,42_ = 10.51, *P* < 0.0001), with decreased *S. equorum* abundance when grown with *P. cyclopium* strain 261 (Dunnett’s test, *P* < 0.0001), *P. cyclopium* strain MB (Dunnett’s test, *P* < 0.0001), and *P. chrysogenum* strain 280 (Dunnett’s test, *P* < 0.0001) compared with control. *P. polonicum* strain 258 slightly reduced *S. equorum* abundance (Dunnett’s test, *P* = 0.092), while *S. equorum* abundance when grown with *P. biforme* strain 277 was comparable with populations grown alone (Dunnett’s test, *P* = 0.99). When excluding extinct populations for normality, *S. equorum* abundance was not significantly different between the treatments, with *S. equorum* abundance only slightly increased in the *P. biforme* strain 277 treatment (Dunnett’s test, *P* = 0.053) and slightly reduced when grown with *P. polonicum* strain 258 (Dunnett’s test, *P* = 0.19), *P. cyclopium* strain 261 (Dunnett’s test, *P* = 0.83), and *P. chrysogenum* strain 280 (Dunnett’s test, *P* = 0.15). *P. cyclopium* strain MB was not included due to *S. equorum* levels below detection in all eight replicate populations (Fig. 2B). In contrast to the variable growth of *S. equorum* across treatments, the five *Penicillium* strains had similar overall levels of growth throughout the 12-week experimental evolution period (ANOVA, *F*_4,35_ = 0.86, *P* = 0.50; Fig. 2C; Table S2). No *Penicillium* populations dropped below detectable levels over the 12-week experimental evolution.

Since several *S. equorum* populations went extinct over the 12-week experimental evolution, we predicted persisting *S. equorum* populations adapted to grow in the selective environments of the *Penicillium* species. To characterize the impact of *Penicillium* on *S. equorum* genome evolution*,* we sequenced the genomes of randomly selected evolved mutant colonies from three replicate populations where *S. equorum* populations persisted over the 12 weeks of the experiment. Within those populations, five colonies were randomly selected for whole-genome sequencing and analysis. These data exclude *P. cyclopium* strain MB populations*,* as all eight replicates contained no detectable bacteria after the 12-week experimental evolution period.

Of the 75 sequenced individual colonies, we observed 645 total (including both synonymous and non-synonymous) mutations, averaging 8.6 mutations per genome (Fig. 2D; Table S3). There were 147 non-synonymous mutations across all isolates, with an average of 1.96 mutations per genome (Fig. 2D; Table S3). We found no statistically significant difference in the number of mutations among all treatments, either in the total number of mutations (ANOVA, *F*_4,70_ = 0.64, *P* = 0.63) or only non-synonymous mutations (ANOVA, *F*_4,70_ = 2.15, *P* = 0.084) (Fig. 2D). There was one outlier, community 25 from growth with *P. biforme* strain 277, in which we detected 25 non-synonymous mutations. We suspect this is driven by mutations in three genes involved in DNA replication and repair, which can result in increased mutation rate via dysfunctional DNA repair mechanisms (49).

When mapped to the reference genome, only 14 genes contained a protein-altering mutation in more than one of 75 sequenced *S. equorum* colonies (Fig. 2E), including three hypothetical proteins with no known function. Of the remaining 11 genes, just two were found to be mutated across independent replicate populations, one of which was found across all five treatments, and another found just in *S. equorum* populations evolved alone. Despite strong effects on mRNA expression in the short-term (Fig. 1) and vastly divergent growth outcomes over the 12 weeks of passaging (Fig. 2A), long-term co-culture with *Penicillium* led to little-to-no genetic change in the genome of persisted *S. equorum* populations (Fig. 2D).

Of the two multi-hit genes found in multiple independent replicates (Fig. 2E), one is a single amino acid mutation in a peptide deformylase found in 51% (38/75) of our sequenced colonies, including in 13/15 independent communities sequenced across all five treatments. All 38 mutants contained a single-nucleotide mutation (C→A), resulting in a leucine to isoleucine mutation at amino acid position 26. It was not found in our re-sequenced wild-type ancestor and found in just over half of our colonies, so it is unlikely due to sequencing error. Peptide deformylases have been well studied as targets of antibacterial inhibitors in both Gram-negative and Gram-positive bacteria (50 - 52). Several groups have studied the effect of single amino acid mutations on enzymatic activity, including in closely related species such as *S. aureus* (53, 54), but none have implicated Leu26 as being a key amino acid. The *S. aureus* peptide deformylase has been crystallized, revealing the conserved Leu26 residue is within a short loop region directly adjacent to an alpha helix on the periphery of the protein structure (54). Leucine and isoleucine are biochemically nearly identical, and thus, a mutation causing a leucine to isoleucine mutation is unlikely to sufficiently alter protein structure or function. As such, it is unlikely the peptide deformylase mutation found in 51% of our experimental evolution mutants can provide a significant increase in mutant fitness compared with its wild-type ancestor.

### Experimentally evolved DHH family phosphoesterase mutations lead to decreased fitness of *Staphylococcus* grown in co-culture with *Penicillium*

The second multi-hit gene encodes a DHH family phosphoesterase, which was mutated in 14 of the 15 sequenced colonies from the evolved alone treatment (Fig. 2E). There were no mutations in this gene detected in any of the *Penicillium* treatments. DHH family phosphoesterases are found in all three domains of life, with the most well-studied orthologs present in humans (Cdc45), *Drosophila* (Prune), and bacteria (RecJ) (55). Through breaking phosphodiester bonds, phosphoesterases can play many different roles in cellular regulation, including in the cleavage of single-stranded DNAs and regulation of di-cyclic mononucleotides, which themselves are common secondary messengers in several signaling pathways (56). The predicted amino acid sequence of the DHH family phosphoesterase gene identified in our study was 82.4% identical to the protein GdpP from *S. aureus* (Uniprot Q2G2T6), a phosphodiesterase responsible for regulating cyclic-di-AMP (CDA) levels. Hence, we will refer to this DHH family phosphoesterase as GdpP and the gene encoding this phosphoesterase as *gdpP*.

**Fig 3:**
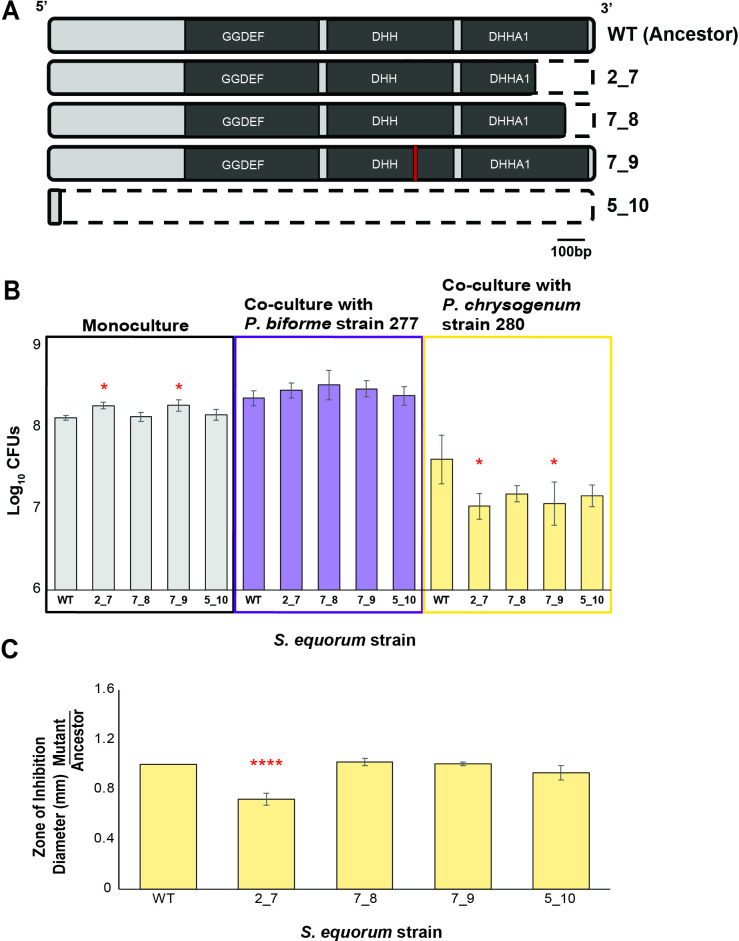
Mutations in a DHH family phosphoesterase gene *gdpP* negatively affect *S. equorum* fitness when grown with antagonistic *Penicillium* strains. Genetically unique *S. equorum* strains (**A**) experimentally evolved alone on cheese for 12 weeks were tested for fitness advantages when grown either in co-culture with *Penicillium* (**B**) or in a solid medium-based spent media experiment (**C**). (**A**) Map of a gene encoding for *S. equorum* DHH family phosphoesterase *gdpP*. Truncated regions are represented by white areas within dashed lines. A H443Y mutation in mutant 7_9 is indicated by the red line. (**B**) Log_10_ total CFUs of each *S. equorum* mutant strain after 7 days of growth on cheese curd agar in monoculture (left), with *P. biforme* strain 277 (middle), and with *P. chrysogenum* strain 280 (right) (*P < 0.05, *N* = 3, *n* = 5). (**C**) Diameters of zone of inhibition (mm) of each *S. equorum* mutant strain after 3 days of growth when grown on plate count agar under a 7-day-old plug of *P. chrysogenum* strain 280 on CCA (****P < 0.0001, *N* = 3, *n* = 3). WT, wild type.

Because *gdpP* mutations were exclusively found when *S. equorum* was evolved alone, we hypothesized that maintaining a functional copy of the *gdpP* gene in *S. equorum* would provide a fitness advantage for the bacteria in co-culture with *Penicillium*. To test our hypothesis, we identified four unique *gdpP* mutations from our 15 sequenced *S. equorum* isolates evolved alone and measured the fitness of a representative isolate of each mutation type in co-culture with *Penicillium* against our ancestral wild-type strain (Fig. 3A). Mutant strain 5_10 contains a single-nucleotide polymorphism (SNP) that results in a truncation event after the third amino acid of a 656-amino acid-long peptide, resulting in a predicted loss of function of the protein (Fig. 3A). Strains 2_7 and 7_8 also contain truncated GdpP proteins, 2_7 in the 583rd of 656 amino acids of the GdpP peptide sequence, and 7_8 in the 619th of 656 amino acids. Finally, mutant strain 7_9 contained a single amino acid substitution, a mutation from a histidine to tyrosine mutation at amino acid position 443, which is the second histidine in the protein’s DHH (aspartate-histidine-histidine) motif (Fig. 3A). These four strains represent each of the four unique GdpP mutants found in our experimental evolution and contained few non-synonymous mutations outside of the *gdpP* coding sequence (Table S3). To experimentally test the fitness of the *gdpP* mutants, we grew the mutants either alone or co-cultured them with one of two functionally different *Penicillium* strains used in this study, *P. biforme* strain 277 which increased *S. equorum* abundance in the evolution experiment and *P.chrysogenum* strain 280 which inhibited *S. equorum*. After 7 days of growth, equivalent to one passage event in our experimental evolution, all four GdpP mutant strains displayed increased fitness in monoculture compared with the ancestral wild-type strain (ANOVA, *F*_4,10_ = 5.59, *P* = 0.016), although this trend was only significant for mutant 2_7 (Dunnett’s test, *P* = 0.023) and mutant 7_9 (Dunnett’s test, *P* = 0.021). In contrast, all four GdpP mutant strains displayed reduced fitness in the presence of *P. chrysogenum* strain 280 compared with the wild-type ancestor (ANOVA, *F*_4,10_ = 3.77, *P* = 0.016; Fig. 3B). Two of the four strains grew significantly worse, mutant 2_7 (Dunnett’s multiple comparison test, *P* = 0.0081) and mutant 7_9 (Dunnett’s test, *P* = 0.011), while mutant 5_10 (Dunnett’s test, *P* = 0.071) and mutant 7_8 (Dunnett’s test, *P* = 0.11) displayed a slight reduction in fitness when grown with *P. chrysogenum* strain 280 compared with the wild-type ancestor. Based on the location of mutations, our data suggest that mutations in the DHH motif in the DHH domain of GdpP, as well as in the C-terminal domain containing the DHHA1 domain, negatively impact *S. equorum* fitness against an antagonistic *P. chrysogenum* strain 280. We did not see a significant difference in fitness between our *S. equorum* mutants when grown with *P. biforme* strain 277 (ANOVA, *F*_4,10_ = 0.88, *P* = 0.51; Fig. 3B), although all four *S*. *equorum* mutants were more abundant in co-culture with *P. biforme* strain 277 than the ancestral wild-type strain.

To better characterize the role of GdpP in *Penicillium–S. equorum* interactions, we created spent cheese medium by incubating *P. chrysogenum* strain 280 on CCA for 7 days, then quantified inhibition by measuring zones of inhibition caused by a plug (1.8 cm diameter) of the spent medium on a lawn of our *S. equorum* mutants. We hypothesize that if GdpP plays a role in defending *Staphylococcus* from inhibitory compounds secreted by *Penicillium*, results would show larger zones of inhibition from our strains with mutated *gdpP* genes. However, we observe the opposite, as the only strain with a significant difference in inhibition is mutant 2_7, in which the mutant displays decreased susceptibility to *P. chrysogenum* strain 280 (Fig. 3C). Therefore, we speculate that GdpP plays a role in *Penicillium–S. equorum* interactions in a mechanism that is likely inducible or contact-mediated.

Previous studies in *S. aureus* have shown loss of function of GdpP confers tolerance to beta-lactams (57 - 59 ), a class of antibiotic secondary metabolites that includes the emblematic penicillin produced by *Penicillium* species. However, we only saw putative loss of function GdpP mutants arise in populations where *S. equorum* was evolved alone, as opposed to in co-culture with *Penicillium* species that can produce beta-lactams (*P. chrysogenum* strain 280 produces the beta-lactam penicillin, based on unpublished observations).

Point mutations in the second histidine residue of the eponymous and conserved DHH motif H443 in *S. aureus* have been shown to result in significantly higher CDA levels (60), and mutant 7_9 contains a mutation at this site. The DHH/DHHA1 domain, where many of our mutations lie, is believed to contain the active site for CDA cleavage (61). We speculate that GdpP confers *S. equorum* a fitness advantage through downstream processes regulated by CDA signaling. Possibilities from biochemical studies in *S. aureus* include ion transport and cell size, as well as receptor protein PstA (61, 62), which is hypothesized to play a role in nitrogen metabolism (63).

### Conclusion

Interactions between *Penicillium* and *Staphylococcus*, as well as fungal–bacterial interactions in general, occupy an important role both historically (4, 5) as well as in current times (64). Relative to the importance of *Penicillium* in medicine, food, and agriculture (3, 30, 65 - 67), our understanding of how *Penicillium* interacts with microbes in their native environments is limited. In our study, we identified both conserved and species-specific transcriptomic responses from our representative bacterial species, *S. equorum* strain BC9, when co-cultured with *Penicillium* (Fig. 1). In both, differentially expressed biochemical pathways and operons all indicate the main impact of *Penicillium* on *S. equorum* expression is nutrient availability, suggesting that *Penicillium–S. equorum* interactions are largely substrate mediated. We also identified both species-level and strain-level diversity in fungal–bacterial interactions. A third of *S. equorum*’s transcriptional response to *P. biforme* strain 277 was unique to that condition alone, despite originating from the same cheese aging facility as three other *Penicillium* strains tested in this study. On the strain level, even two strains of the same species identity (*P. cyclopium* strain 261 and strain MB), despite eliciting similar transcriptional responses (Fig. 1), had divergent impacts on *S. equorum* long-term growth in co-culture experimental evolution (Fig. 2B).

Despite a vast transcriptomic response to *Penicillium* (Fig. 1C) and evidence of *Penicillium* driving *S. equorum* to extinction in co-culture, long-term passaging of *S. equorum* in co-culture did not consistently impact genome evolution of *S. equorum* (Fig. 2D). The only evidence of parallel evolution we identified was found in bacterial populations evolved alone without a *Penicillium* species (Fig. 2E). From this, we identified a DHH phosphoesterase as putatively involved in *Penicillium–S. equorum* interactions on CCA (Fig. 3), although with limited information regarding the biological function of the protein, it is difficult to speculate a mechanism of action beyond GdpP’s likely role in regulating cytosolic cyclic-di-AMP levels (56, 61).

While all our synthetic communities consisted of two species (one bacterium and one fungus), cheese rind microbiomes typically contain around five to seven different species (22). As such, our study does not address higher-order interactions that could affect both mRNA expression and evolution outcomes (68). However, outcomes of pairwise interactions, such as those found in our study, have been able to predict community assembly patterns (69). Furthermore, because we passaged the *Penicillium* along with our bacterium of interest *S. equorum*, we cannot rule out *Penicillium* evolving over the 12-week experimental evolution as an influencing factor in our results. Previous work in the lab has shown that a *P. biforme* strain closely related to this study’s *P. biforme* strain 277 produced fewer pigments, spores, and secondary metabolites after just four weeks of passaging on CCA (70), and these phenotypes could affect how *Penicillium* interacts with bacteria. However, by allowing both our bacterial strain of interest as well as co-cultured *Penicillium* to evolve, our experimental evolution more closely resembles cheese production facilities where both species are allowed to evolve during the aging process.

It is possible that the limited evolution that we observed in our *S. equorum* populations is because of the number of generations over the period of our study. We estimate approximately 180–210 *S. equorum* generations occurred within our 12-week experimental evolution, depending on the *Penicillium* treatment. A previous study from our lab studying a similar *Staphylococcus* species evolved for a longer period (450 generations) but identified both genetic and phenotypic variations within evolved populations by 150 generations (24). Furthermore, *S. equorum* showed clear evidence of evolution in monoculture in the same period of time and under parallel experimental conditions, which suggests that our particular fungal–bacterial combination has constrained genome evolution of *S. equorum*. It is also possible, particularly in the case of populations that were strongly inhibited by *Penicillium* species, that genetic variation we see is due to genetic drift as a result of low population sizes. However, in monoculture, where we identify the only strong evidence of parallel evolution, we transferred roughly 300,000 colony-forming units (CFUs) per passage, reducing the impact of drift. Because we identified four unique mutations within the *gdpP* gene coding sequence in 14/15 replicate colonies obtained from three independent communities, we are confident that this particular mutation we observed is not merely due to genetic drift or stochastic effects. Future evolution experiments over a longer period of time and with different conditions (different strains of *S. equorum*, different population bottleneck sizes, and additional biotic complexity with more microbial species) will more comprehensively characterize the potential for *S. equorum* to adapt to different biotic environments on cheese.

Despite attempts by our lab and others, genetic tools for constructing *de novo* mutants are not yet available for *S. equorum*, so we were unable to make “clean” *gdpP* mutations that we observed in the monoculture-evolved populations. While we chose evolved mutant strains with the fewest number of secondary non-synonymous mutations in the genome (Fig. 3), three of the four mutant strains chosen had one to two additional non-synonymous mutations. Mutant 2_7 contains the peptide deformylase mutation described above, while mutant 7_8 contains a single amino acid mutation in a predicted coding sequence of unknown function. Mutant 5_10 contained two additional non-synonymous mutations, a truncation event in a cell envelope-associated transcriptional attenuator gene and a single amino acid mutation in a gene encoding for sorbitol dehydrogenase. We cannot rule out the potential effects of these non-synonymous mutations on strain fitness. Nevertheless, we were able to measure decreased fitness in multiple experimental evolution descendants with a mutation in this DHH phosphoesterase gene, including mutant 7_9, which has no other non-synonymous mutations within its genome (Fig. 3B).

Filamentous fungi can produce antimicrobial secondary metabolites that are thought to inhibit bacteria in natural environments (71). Two of the fungi in our experiments are known to be antibacterial: we have observed *P. chrysogenum* strain 280 producing penicillin in the lab (unpublished data) and have observed strong antibacterial activity of *P. cyclopium* strain MB (25). Because of their potential to inhibit bacteria, we predicted that the transcriptional responses would reflect growth in stressful environments where they needed to tolerate the presence of the fungi. Surprisingly, the majority of *S. equorum*’s transcriptional response was nutrient based rather than related to defense or antibiotic tolerance. Of all enriched KEGG pathways, just the *opp* operon, upregulated only when co-cultured with *P. biforme*, is involved in defense. It is possible that this response is specific to cheese, as filamentous fungi are speculated to produce fewer secondary metabolites when grown on cheese (72, 73). Alternatively, we may have measured mRNA expression at a time where *S. equorum* and/or *Penicillium* species are prioritizing growth over direct competition. To this effect, future studies should consider the potential effects of temporal variation on *Penicillium–S. equorum* interactions. Nevertheless, we show evidence of a complex relationship between *Penicillium* and *S. equorum*, in which both inhibitory and beneficial interactions are conflicting forces that affect population dynamics. This complexity can be seen most clearly in the relationship between *P. chrysogenum and S. equorum*, where between 6 and 12 weeks of evolution, *S. equorum* populations in co-culture with *P. chrysogenum* strain 280 were either higher compared with growth alone or below detectable limits (Fig. 2B). Similarly, *S. equorum* abundance was higher in co-culture with *P. chrysogenum* strain 280 compared with alone after 3 days of growth (Fig. 1B) but lower after 7 days of growth (Fig. 3B).

An ongoing question in our study is the disparity between the effect of *Penicillium* on *S. equorum* transient expression compared with longer-term genetic evolution. A potential explanation is that this particular strain of *S. equorum* (BC9) may have already evolved to become well adapted to the cheese rind environment and to growing with *Penicillium*. Alternatively, either we did not carry out the experimental evolution study long enough to capture genetic mutation, or the mechanisms of interactions between *Penicillium* and *S. equorum* temporally varied, such that selective pressure was not consistently maintained through the 12-week period.

While our study was limited to pairwise interactions, we can apply our findings to better understand more complex cheese and non-cheese microbiomes. *S. equorum* is a widespread and critical component of many surface-ripened cheeses and fermented meats (15, 32). Our findings suggest the species of fungi grown with *S. equorum* can impact the bacteria’s short-term and long-term biology, which can then in turn affect cheese quality and safety (74). Furthermore, our study identified conserved and species-specific responses to fungal growth that align with other fungal–bacterial interactions studies which have highlighted iron and B vitamins as key molecules involved in fungal–bacterial interactions in cheese microbiomes (15, 18) and beyond (40 - 42). Finally, *S. equorum* is a non-pathogenic relative of the well-studied pathogen *S. aureus* (75 ). While we do not know if *S. equorum*–fungal interaction mechanisms will translate to *S. aureus*, our findings provide a foundation for studying *Staphylococcus*–fungal interactions in a broad range of microbial systems.

## MATERIALS AND METHODS

### Strains and growth conditions

All strains used in this study were isolated from cheese rinds aged in various cheese aging facilities across USA and characterized by 16S rRNA or ITS gene sequencing (22). *S. equorum* strain BC9 has been previously characterized in the lab (22), as has *P. cyclopium* strain MB (25). The remaining four *Penicillium* strains used in this study, *P. polonicum* strain 258, *P. cyclopium* strain 261, *P. biforme/commune* strain 277, and *P. chrysogenum* strain 280 were isolated from the same cheese aging facility by our lab prior to this study (unpublished data).

### Growth conditions

*S. equorum* strains were frozen at −80°C in 15% glycerol in brain–heart infusion (BHI) liquid media. *Penicillium* strains were harvested as spores from dense streaks on plate count agar with milk and salt (PCAMS; 22), and frozen at −80°C in 15% glycerol in 1× phosphate-buffered saline (1× PBS). For all experiments, CFU counts were determined by serially diluting frozen glycerol cultures on PCAMS. All experiments were carried out on CCA (22) at 24°C, with fungal and bacterial abundance determined by serially diluting in 1× PBS and plating on PCAMS with chloramphenicol (50 mg/L) and PCAMS with natamycin (21.6 mg/L) respectively, as previously described (76).

### RNA extraction and sequencing

To obtain bacterial cultures for RNA extractions, we co-inoculated 20,000 CFUs of *S. equorum* strain BC9 onto 100 mm petri dishes containing 20 mL CCA with 20,000 CFUs of one of five *Penicillium* species tested in this study. For a control (no fungus) treatment, we replaced the *Penicillium* inoculum with an equivalent volume of 1× PBS. After 72 hours of undisturbed growth in a dark incubator set to 24°C, cells were scraped off the agar surface with an ethanol-sterilized razor blade and frozen at −80°C submersed in RNAProtect Bacteria Reagent (Qiagen, Germany). Following at least 24 hours at −80°C, cells were removed from RNAProtect and incubated at 37°C for 15 minutes in 200 µL of Tris-EDTA buffer (50 mM Tris-HCl, 50 mM EDTA, pH 8) containing lysozyme (50 mg/mL), lysostaphin (22 U/mL), and Triton X-100 (1.2%) to ensure staphylococcal cell wall disruption. RNA was then extracted from the lysis solution with 125:24:1 (vol/vol/vol) phenol/chloroform/isoamyl alcohol, as previously described (16). There were three biological replicates per treatment.

Residual DNA was removed by incubation with DNase (Qiagen, Germany) followed by column purification with RNA Clean & Concentrator Kit-5 (Zymo Research, Irvine, CA, USA). RNA samples were confirmed to be DNA-free by the absence of DNA bands on a 1% agarose gel following amplification of the bacterial 16S region by PCR. Bacterial rRNA was depleted using a NEBNext rRNA Depletion Kit (Bacteria) (New England Biolabs, Ipswich, MA, USA). Fungal rRNA was depleted by incorporating a custom-designed pool of complementary *Penicillium* rRNA sequences (Table S4) with the NEBNext RNA Depletion Core Reagent Set (New England Biolabs, Ipswich, MA, USA) in accordance with kit instructions. Fungal rRNA depletion probe pool was diluted to a final concentration of 2 µM (for each probe) in TE Buffer (10 mM Tris, 0.1 mM EDTA, and pH 7.5) prior to addition. Depleted RNA was then prepped for Illumina sequencing with NEBNext Ultra II RNA Library Prep Kit for Illumina following NEB protocol instructions for preparation of intact RNA (New England Biolabs, Ipswich, MA, USA), then pooled and sequenced at Tufts University Core Facility Genomics (Boston, MA, USA) on a NextSeq 550 (Mid-output, 150 cycles).

### Differential expression analysis

Raw Illumina reads were mapped to an assembled *S. equorum* BC9 genome (NCBI WGS Accession #LNNB00000000) using Geneious Prime mapper (version 2020.2.5) at medium-low sensitivity, with reads mapping to multiple best matches randomly assigned. Differential expression was calculated with DESeq2 (77) with triplicate replicates grouped. Genes were considered differentially expressed if expression when grown with *Penicillium* species in CCA was greater than doubled (> log_2_ ratio of 1) or less than halved (< log_2_ ratio of −1) compared with growth alone on CCA at a *P*-value < 0.05, adjusted for false discovery rate using the Benjamini–Hochberg procedure (77). To identify pathways that were enriched in differentially expressed genes, we used the KOBAS-i Gene-list Enrichment tool (34) with our BC9 genome as the background genes to test for enrichment.

### Experimental evolution

About 200 CFUs of BC9 were inoculated in 1.5-mL microcentrifuge tubes (USA Scientific, Ocala, FL, USA) containing 150 µL CCA alone or with 200 CFUs (10 µL at 20 CFUs/µL) of one of five *Penicillium* species tested in this study. Samples were incubated at 24°C undisturbed in the dark for 7 days. After 7 days, samples were diluted with 300 µL 30% glycerol in 1× PBS to a final concentration of 10% glycerol in 1× PBS and homogenized by pestling. About 10 µL (2%) of the homogenized sample was inoculated into a fresh microcentrifuge tube containing CCA. This process was subsequently repeated every 7 days for 12 weeks. Every 21 days, homogenized samples were serially diluted in 1× PBS and plated on both PCAMS with chloramphenicol (50 mg/L) and PCAMS with natamycin (21.6 mg/L) to access *Penicillium* and *S. equorum* abundance, respectively.

After 12 weeks of evolution, samples were homogenized by pestling, serially diluted in 1× PBS, and plated on PCAMS containing natamycin (21.6 mg/L). After 48 hours of growth, five S. *equorum* colonies randomly selected from three randomly selected surviving replicate communities for each of five treatments (excluding MB treatment where BC9 went extinct in all eight replicates) were inoculated in 4 mL BHI liquid media. After 24 hours of growth in a shaking tabletop incubator (450 rpm, room temperature), 500 µL of the overnight culture was diluted 1:1 in 30% glycerol in 1× PBS and frozen at −80°C for mutant fitness comparison experiments (see below). The remainder was pelleted to remove media supernatant, washed in 1× PBS, and frozen at −20°C for whole-genome sequencing.

### Whole-genome sequencing and variant analysis

DNA was extracted from a pelleted cell culture following DNeasy Blood and Tissue Kit’s protocol for pretreatment for Gram-positive bacteria (Qiagen, Germany). To ensure lysis of *Staphylococcus* cells, the enzymatic lysis buffer was supplemented with lysostaphin (22 U/mL). DNA was then sequenced on a NextSeq 2000 at the Microbial Genome Sequencing Center (MiGS, Pennsylvania, PA, USA) at a minimum depth of 200 MBp.

Raw reads from sequencing were mapped to an assembled BC9 genome using Geneious mapper at medium-low sensitivity, with reads mapping to multiple best matches randomly assigned. Variants were found using Geneious’s Find Variations/SNPs tool with a minimum coverage of 20 and a minimum variant frequency of 0.9. Both SNPs and small insertions and deletions were identified. Two mutations were found in all 76 genomes (75 descendants and 1 ancestral strain), including a non-synonymous mutation in a truncated biofilm-associated surface protein and thus were considered sequencing errors and removed from the analysis. A single-nucleotide transversion mutation was also found in the ancestral strain but was kept in the calculations as the mutation only appeared in 52 of the 75 descendant genomes. Eight evolved genomes contained a missing adenine in a 10-nucleotide tandem repeat; this mutation was not found in the ancestral strain and thus was kept into consideration. Neither of the latter two mutations were found within an open reading frame.

### Mutant fitness comparison

From our original set of 15 colonies of BC9 strains evolved alone on CCA, 4 genetically unique *S. equorum* strains were selected for mutant fitness experiments. All non-synonymous mutations found in each strain are listed in Table S3.

For co-culture experiments, each BC9 mutant along with a wild-type ancestral control was inoculated into 1.5-mL microcentrifuge tubes (USA Scientific, Ocala, FL, USA) containing 150 µL CCA alone or with 200 CFUs of either *P. biforme* strain 277 or *P. chrysogenum* strain 280. The communities were allowed to incubate at 24°C in a dark incubator for 7 days, then diluted 1:5 in 15% glycerol in PBS, homogenized by pestling, and plated out on both PCAMS with chloramphenicol (50 mg/L) and PCAMS with natamycin (21.6 mg/L) to access abundance of *Penicillium* species and *S. equorum*, respectively. Experiments were completed in triplicate, with five experimental replicates per biological replicate.

For plug-on-lawn experiments, *Penicillium* strains were densely inoculated onto CCA agar at 500 CFUs/µL and incubated at 24°C in a dark incubator for 7 days. A cork borer (18 mm diameter) was then used to punch out a circular plug (18 mm diameter) of the spent media. The plug was carefully placed, spore side up, on a PCAMS + natamycin (21.6 mg/L) plate previously inoculated with 100 µL of an overnight liquid culture in BHI (shaking at 450 rpm, room temperature) of either one of five BC9 mutants or a wild-type control diluted to an OD_600_ of 0.01 in 1× PBS. Plates were incubated at 24°C for 3 days before measuring the zone of inhibition diameters.

### Statistical analysis

Standard statistical analyses were conducted in JMP Pro (version 16.2.0). Quantitative data were log transformed and analyzed with one-way ANOVA parametric tests. If significance was detected via ANOVA tests, post hoc Dunnett’s tests were conducted. All data were calculated with the means of experimental replicates. *N* values refer to the number of independent experimental replicates conducted over separate occasions with separate materials and cultures. *n* alues refer to the number of independent biological replicates within each experimental replicate.

## Data Availability

All raw Illumina reads of sequenced evolved isolates of *S. equorum* (BC9) have been deposited in NCBI in BioProject PRJNA922588. Raw Illumina reads from RNA-sequencing have been deposited in NCBI in BioProject PRJNA922923.

## SUPPLEMENTAL MATERIAL

Table S1 msphere.00047-23-s0001.xlsx Differential mRNA expression with accompanying *P*-values in *S. equorum* in response to *Penicillium* species.

Supplemental Data msphere.00047-23-s0002.pdf Fig. S1, Fig. S2, and Tables S2 and S4.

Table S3 msphere.00047-23-s0003.xlsx All genomic mutations found in *S. equorum* colonies evolved for 12 weeks on cheese curd agar and their predicted effect on protein sequence, if any.

ASM does not own the copyrights to Supplemental Material that may be linked to, or accessed through, an article. The authors have granted ASM a non-exclusive, world-wide license to publish the Supplemental Material files. Please contact the corresponding author directly for reuse.
