# Supplementary material for: Penicillium molds impact the transcriptome and evolution of the cheese bacterium Staphylococcus equorum
Source: mSphere. 2023 May 23;8(4):e00047-23. doi: 10.1128/msphere.00047-23 (PMC10449494; doi:10.1128/msphere.00047-23)
Supplement: Supplemental Data — Fig. S1, Fig. S2, and Tables S2 and S4. [file msphere.00047-23-s0002.pdf]

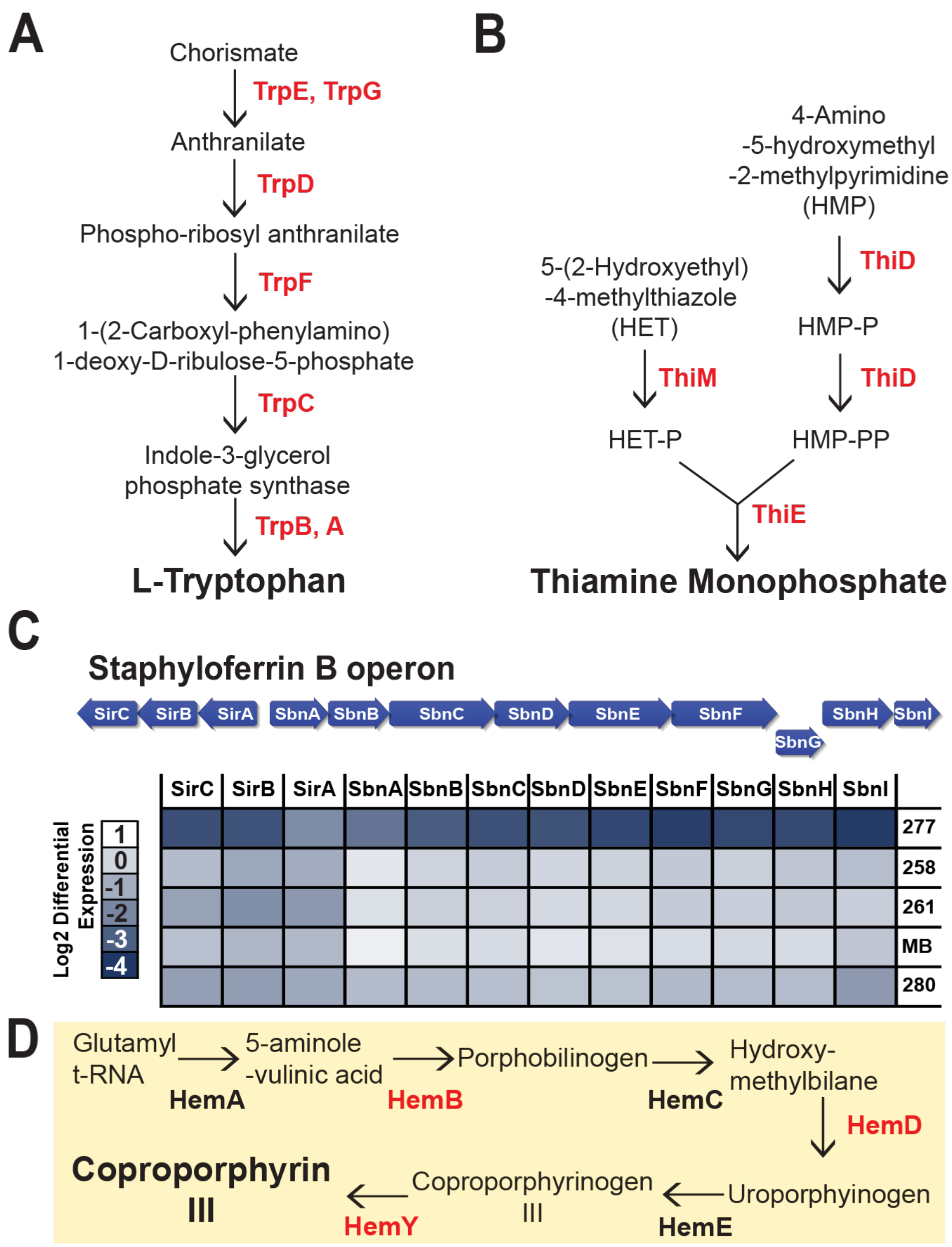

Figure S1. Differentially expressed pathways in *S. equorum* when grown with *Penicillium* species. Differential mRNA expression in *Staphylococcus equorum* after 72h of growth with one of five *Penicillium* strains compared to growth alone. (n = 3, P < 0.05). Significant differential expression is defined as fold change greater than $\log_2 1$ or less than $\log_2 -1$ when grown with *Penicillium* compared to growth in monoculture. Biosynthesis pathways of L-tryptophan (**A**) and thiamine monophosphate **(B)**, with enzymes in red indicating a gene that is significantly upregulated in all *Penicillium* treatments. **(C)** Gene map of *S. equorum* in staphyloferrin B operon, with downregulated genes in blue. Increasing color intensity corresponds with stronger downregulation in co-culture compared to *S. equorum* grown alone. **(D)** Biosynthesis pathway of coproporphyrin III in *S. equorum*, with enzymes in red indicating a gene that is significantly upregulated only when grown with *P. chrysogenum* strain 280.

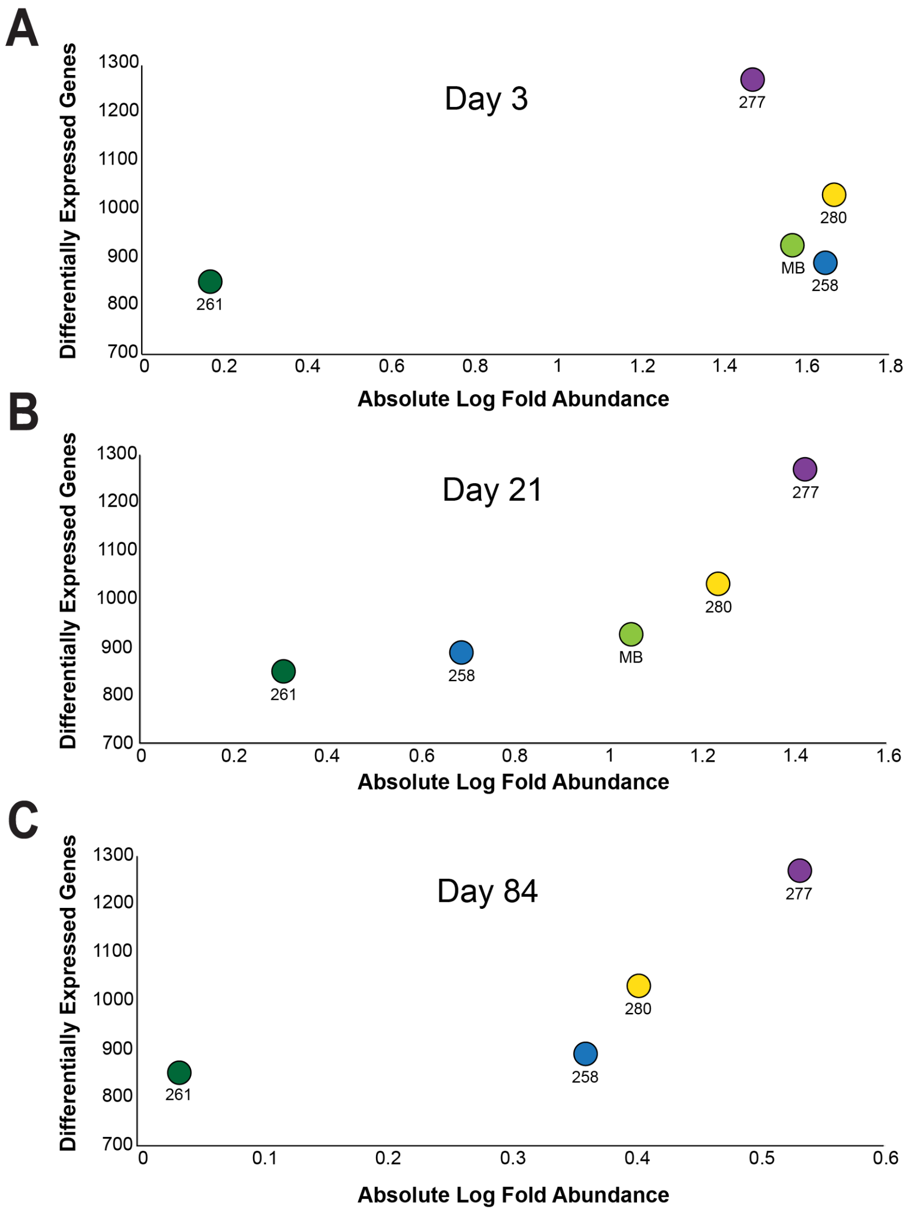

Figure S2. *S. equorum* transcriptomic response to *Penicillium* relative to bacterial abundance in co-culture. Differential mRNA expression in *Staphylococcus equorum* plotted against change in abundance when grown with one of five *Penicillium* species compared to growth alone. Total number of differentially expressed genes is defined as the number of genes within the *S. equorum* genome with a fold change greater than $\log_2 1$ or less than $\log_2 -1$ when grown with *Penicillium* compared to growth in monoculture for 72h (n = 3, P < 0.05). Abundance is calculated as absolute log fold change in total CFUs after 72h of growth in parallel to mRNA extraction (N=3, n=5) (**A**), after 3 weeks of experimental evolution (**B**), or after 12 weeks of experimental evolution (**C**). Data for abundances were calculated from data represented in **Fig. 1B** (**A**), **Fig. 2B** (**B**), and **Fig. 2C** (**C**). Data for differential mRNA expression (y-axes) are represented in **Fig. 1C.** For normality, experimental evolution populations where *S. equorum* was no longer detectable were excluded in calculations. Points on the graph represent each *Penicillium* treatment except for Day 84 co-cultured with *Penicillium cyclopium* strain MB, where *S. equorum* went below detectable limits in all eight replicate populations. Points on the graph are colored according to the *Penicillium* treatment color scheme in **Fig. 1** and **Fig. 2**.

Table S2. Abundance of *S. equorum* and *Penicillium* species over a twelve-week experimental evolution on cheese curd agar, passaged weekly and measured for microbial abundance every three weeks.

		BC9 (CFUs/mL)				Penicillium (CFUs/mL)			
**Neighbor**	**Population**	**Week 3**	**Week 6**	**Week 9**	**Week 12**	**Week 3**	**Week 6**	**Week 9**	**Week 12**
None	1	18000000	12000000	38000000	65000000	0	0	0	0
None	2	6000000	14000000	9200000	14600000	0	0	0	0
None	3	36000000	56000000	56000000	34000000	0	0	0	0
None	4	14000000	52000000	30000000	31000000	0	0	0	0
None	5	28000000	28000000	22000000	42000000	0	0	0	0
None	6	22000000	38000000	42000000	32000000	0	0	0	0
None	7	42000000	40000000	58000000	53000000	0	0	0	0
None	8	26000000	14000000	24000000	16800000	0	0	0	0
P. 258	9	0	0	0	0	1740000000	1080000000	109000000	107000000
P. 258	10	3000000	50000000	9400000	10900000	1380000000	1880000000	213000000	67000000
P. 258	11	0	0	0	12900000	1920000000	1420000000	55000000	14500000
P. 258	12	0	0	0	0	3840000000	1940000000	66000000	50000000
P. 258	13	0	0	0	0	720000000	1300000000	61000000	57000000
P. 258	14	5400000	78000000	580000	26000000	1220000000	1020000000	46000000	82000000
P. 258	15	0	0	0	27000000	1840000000	1580000000	59000000	75000000
P. 258	16	6400000	36000000	380000	1900000	1700000000	2300000000	56000000	36000000
P. 261	17	44000000	740000000	98000000	41000000	1020000000	640000000	113000000	70000000
P. 261	18	0	0	0	0	700000000	720000000	103000000	38000000
P. 261	19	78000000	660000000	128000000	67000000	1200000000	1060000000	99000000	92000000
P. 261	20	32000000	700000000	46000000	2500000	1020000000	880000000	77000000	41000000
P. 261	21	40000000	560000000	100000000	23000000	920000000	1280000000	97000000	36000000
P. 261	22	0	0	0	0	900000000	1100000000	118000000	80000000
P. 261	23	0	0	0	0	1140000000	1200000000	43000000	100000000
P. 261	24	0	0	0	0	720000000	3160000000	110000000	92000000
P. 277	25	680000000	580000000	116000000	122000000	580000000	400000000	34000000	11400000
P. 277	26	800000000	900000000	132000000	162000000	560000000	420000000	56000000	13100000
P. 277	27	680000000	460000000	120000000	45000000	800000000	640000000	13000000	10300000
P. 277	28	720000000	700000000	440000000	74000000	780000000	680000000	50000000	12000000
P. 277	29	700000000	680000000	240000000	55000000	680000000	740000000	23000000	7600000
P. 277	30	500000000	260000000	500000000	115000000	440000000	520000000	41000000	3600000
P. 277	31	380000000	760000000	520000000	234000000	354000000	680000000	44000000	20200000
P. 277	32	640000000	320000000	780000000	174000000	420000000	1220000000	36000000	4000000
P. 280	33	360000000	50000000	4800000	0	1000000000	980000000	55000000	53000000
P. 280	34	440000000	0	0	0	960000000	760000000	52000000	31000000
P. 280	35	700000000	14000	0	0	1760000000	960000000	55000000	28000000
P. 280	36	280000000	60000000	0	0	840000000	700000000	39000000	52000000
P. 280	37	140000000	98000000	88000000	28000000	1140000000	820000000	38000000	18500000
P. 280	38	460000000	12000	0	0	1100000000	880000000	33000000	19900000
P. 280	39	480000000	36000000	36000000	12900000	820000000	1200000000	75000000	35000000
P. 280	40	460000000	90000000	40000000	1900000	1700000000	500000000	13400000	6500000
P. MB	41	0	0	0	0	1840000000	840000000	92000000	103000000
P. MB	42	0	0	0	0	1380000000	1260000000	178000000	78000000
P. MB	43	0	0	0	0	1860000000	620000000	34000000	559000000
P. MB	44	2000000	14000000	6000	0	1240000000	1220000000	98000000	60000000
P. MB	45	0	6000	0	0	2400000000	4260000000	93000000	70000000
P. MB	46	2600000	10000	0	0	1340000000	1360000000	256000000	92000000
P. MB	47	0	0	0	0	1300000000	5160000000	82000000	101000000
P. MB	48	1800000	80000	0	0	1020000000	1220000000	97000000	76000000

Table S4. Custom designed pool of complementary *Penicillium* rRNA sequences.

Pen_rRNA_Pool Sequences (5' to 3')
TGAGCGGACGGGAAGCCCTATTTTCCACACCCTATGGTCG
ACCCACCCAACTACTAACTCACCGGCGTGTGGCTTGAGTA
AACTTTGATTTCTCGTAAGGTGCCGAACGGGTCATTATAG
GTTGAGTCAAATTAAGCCGCAGGCTCCACGCCTTGTGGTG
CTTAAGAAGCCAGCGGCCCGCAAATGCGGACCGGGCTATTTAAGGGCCGAGGTC
GCTTGAGCCGATAGTCCCCTTAAGAAGCCAGCGGCCCGCA
GAAGCATCCTCTGCAAATTACAATGCGGACCCCGAAGGAGCCAGCTTTCAAATTT
TCTATGACGTCCCGTTCCAGGGCACTTAGATGGGGACCGCTCCCGAAGCATCCTCT
CATCCCATACGGGATTCTCACCCTCTATGACGTCCCGTTC
ATTCCCAAACAACTCGACTCGTCGAAGGAGCTTCACACGGGCGCGGACACCCCAT
AGCTTTAGATGAAATTTACCACCCATTTAGAGCTGCATTCCCAAACAACT
CTACTTGTGCGCTATCGGTCTCCGGCCAATATTTAGCTTTAGATGAAATT
CTTTTCATCTTTCGATCACTCTACTTGTGCGCTATCGGTC
GCGCTTCCCTTTCAACAATTTCACGTGCTTTTTAACTCTCTTTTCAAAGTG
GCGAGCGAGTCTGGTCGCAAGCGCTTCCCTTTCAACAATT
GACGCTGGCCCGCCCGCGGGGAAGTACACCGGCACGAATGCCGGCTGAACCCCGC
ACCGACCGCCCAAACCGACGCTGGCCCGCCCGCGGGGAAG
CTATAAGACGCCCCTAGGGGCGTTACCTTCCGAGGGCCTTTGACCGAC
GTTCCTCGGTCTAGGCAGGTCGCATTGCACCCTCGGCTATAAG
ATTATGCCAGCGTCCGAGCCGAAGCGCGTTCCTCGGTCTA
TAGATGTTAGACTCCTTGGTCCGTGTTTCAAGACGGGTCGCTTACGACCATTAT
GTTTGACACCCGAACACTCGCGTAGATGTTAGACTCCTTG
TCGATGGTGCACCCGTAAGGGTTCCCACCTCCGTTCGCTTTCACTTCGCGCA
CCGAAGACTTCAGGATCGGTCGATGGTGCACCCGTAAGGG
CCCAACAGCTACGCTCTTACTCAAATCCATCCGAAGACTT
CCTATTCAGGCATAGTTCACCATCTTTCGGGTCCCAACAG
ACGTCAGAACCGCTACGAGCCTCCACCAGAGTTTCCTCTGGCTTCGCCCT
CAAATTCGACGATCGATTTGCACGTCAGAACCGCTACGAG
CAGGAACCAGCTACCAGATGGTTCGATTAGTCTTTCGCCCCTATACCCAAA
TATCCTGAGGGAAACTTCGGCAGGAACCAGCTACCAGATG
CCAAGGCCTCTAATCATTCGCTTTACCTCATAAAACTGAATTCGCGTTAC
ACATATTTAAAGTTTGAGAATAGGTTAAGGTTGTTTCAACCCCAAGGCC
ATGCCCACGTTCAATTAAGCAACAAGGGCTTCTTACATATTTAAAGTTTG
CACTAGTAACGCATCATTCTAATGCCCACGTTCAATTAAG
CTTAACCTCGCGTTCGGTTCATCCCGCATCGCCAGTTCTGCTTACCAAAAAT
CTGTCTAGATGAACTAACACCTTTTGTGGTGTCTGATGAGCGTGTATTCC
GATTCCGACTTCCATGGCCACCGTCGGGCTGTCTAGATGAACTAACAC
GTGAGTTGTTACACACTCCTTAGCGGATTCCGACTTCCATG
TCCAACTGCTGTTCACATGGAACCTTTCCCCACTTCAGTCCTCAAAGTT
AGCCGGGCTTGAGGGCAGCAATGACGCTCGGACAGGCATGCCCCCCGGAATACCAGGG
GGGCCCAACACACAAGCCGGGCTTGAGGGCAGCAATGACG
TTTCGGGCCCGTCCCCCGGAAATCGGAGGACGGGGCCCAA

ATTTAGAGCTGCATTCCCAAACAACTCGACTCGTCGAAGGAGCTTCACAC
CGGTCTCCGGCCAATATTTAGCTTTAGATGAAATTTACCACCCATTTAGAG
TCACTCTACTTGTGCGCTATCGGTCTCCGGCCAATATTTA
ACTCTCTTTTCAAAGTGCTTTTCATCTTTCGATCACTCTACT
GAGTCTGGTCGCAAGCGCTTCCCTTTCAACAATTTCACGTGCTTTTTAA
CGCGGGGAAATACACCGGCACGAATGCCGGCTGAACCCCGCGAGCGAGTCTGGTCGC
CGACGCTGGCCCGCCCGCGGGGAAATACACCGGCACGAAT
TAGGGGCGTTACCTTCCGAGGGCCTTTGACCGACCGCCCAAACCGACGCT
TCCTCGGTCTAGGCAGGTCGCATTGCACCCTCGGCTATAAGACGCCCCTAGGGGCGT
GAGCCGAAGCGCGTTCCTCGGTCTAGGCAGGTCGCATTGC
TTGGTCCGTGTTTCAAGACGGGTCGCTTACGACCATTATGCCAGCGTCCGAGCCGAA
GAACACTCGCGTAGATGTTAGACTCCTTGGTCCGTGTTTCAAGAC
TAAGGGTTCCCACCTCCGTTCGCTTTCACTTCGCGCACGGGTTTGACACCCGA
TCGGTCGATGGTGCACCCGTAAGGGTTCCCACCTCCGTTC
CTACGCTCTTACTCAAATCCATCCGAAGACTTCAGGATCGGTCGATG
CACCATCTTTCGGGTCCCAACAGCTACGCTCTTACTCAAA
ACCCACCCAACTACTAACTCACCGGCGTGTGGCTTAAGTA
CACCAGACTTGCCCTCCAATTGTTCCTCGTTAAGGGATTT
TTTAACTGCAACAACTTTAATATACGCTATTGGAGCTGGAATTACCGCGG
CGGACCGGCCAGCCAGGCCCAAGGTTCAACTACGAGCTTTTTAACTGCAACA
CAACACACAAGCCGTGCTTGAGGGCAGCAATGACGCTCGGACAGGCATGCC
GCCCGTCCCCCGGAATCGGAGGACGGGGCCCAACACACAAGCCGTGCTTG
GGTGCCGCCGCTGCCTTTCGGGCCCGTCCCCCGGAATCGG
CTACAGAGCGGGTGACAAAGCCCCATACGCTCGAGGACCG
TATCCCTACCTGATCCGAGGTCAACCTGGATAAAAATTTGGGTTGATCGGCAAGCG
TTATTGATATGCTTAAGTTCAGCGGGTATCCCTACCTGATCCGAGGTCAA
GGCAATCCCTGTTGGTTTCTTTTCCTCCGCTTATTGATATGCTTAA
CTTTCAAATTTGAGCTCTTGCCGCTTCACTCGCCGTTACTG
GGATCACTCCCAAGGCTTCGTCACGGACCCCCACGCCTGC
ACCACCAAGATCTGCACTAGGGGCCGTTCGACCCGGGATC
CCACTTCAGTCCTCAAAGTTCTCATTTGAGTATTTGCTACTACCACCAAG
TAACCCATGTCCAACTGCTGTTCACATGGAACCTTTCCCCACTTCAGTCCTCAAAGT
AAACGGAACTTCCCTATGCCTTAGGATCGACTAACCCATGTCCAACTGCTGTTCAC
TCGGCAAACGGCGCACGAGGGCGCCTTTAAAACGGAACTTCCCTATGCCTTAG
TGTTAACCGGCTTCCCTTTCGGCAAACGGCGCACGAGGGC
CGCCGACGTCTCCGCGTTCAGTTACGTTGCCGTGGAGAATCCACATCTAGGTGCCGGA
CTTCCCAGGACCCCCGCCGACGTCTCCGCGTTCAGTTACG
TCAGGGTGATAGGCTGTCAAGAAGAAAAGAGAACTCTTCCCAGGA
GCAAAAGTGCGGCGCTCTGCCAGCCATAGAACCCTAGCTCCGGACAAACCGATTTCAGG
CTTCCCGCGGATTTTCAAGGGTCGTCGGGGGCGCACCGGA
CTTGGAGACCTGCTGCGGTTATGAGTACGACCTAGCGTGAAAACTATTC
TTATCTACATTGTTCTATCAACTAGAGGCTGTTCACCTTGGAGACCTGCT
CCGAAGTTACGGATCCATTTTGCCGACTTCCCTTATCTACATTGTTCTAT
CCGACCCTTAGAGCCAATCCTTATCCCGAAGTTACGGATC
GCCCGGCTAGTGCCCTACTGCTCCAGGAGGTTCCCCCCAAGGCCCAGCGAGCCCGA

GGGCGTCCCCAACCCGGTGCTGCGGGGCGCCGGCCGGTTGCCCGGCTAGTGCC
ACGGCCGAAGCCTGCCAAGGGCGTCCCCAACCCGGTGCTG
CAGTTCTAAGTTGGTCGTTAATCGCCCGCCGGACGGCCGA
ATCGCAATGCTATGTTTTAATTAGACAGTCAGATTCCCCTTGTCCGTACCA
GATCGGCCCGCGAAGGACCTTAACGCCAGAAGATGGGCGGTGAAGCCCAGTTCCGC
CTGACAATGTCTTCAACCCGGATCGGCCCGCGAAGGACCTTAAC
ACACCTGCGTTGTTGTTTAACAGATGTGCCGCCCCAGCCAAACTCCCCACCTGACAA
GAGATTTCTGTTCTCCATGAGTCCCCCTTAGGACACCTGCGTTGTTGTTTAACAGAT
CCCTTTTGTTCTACTGGAGATTTCTGTTCTCCATGAGTCCC
TGGTTTGTATTCACACTGAAAATCAAAATCAAGGGGACTTTTACCCTTTTGT
GGACTAAAGGATCGATAGGCCACACTTTCATGGTTTGTATTCACACTGAA
ACTTTTCTGGCACCTCTAGCCTCAAATTTCGAGGGACTAAAGGATCGATA
CTATGAACGCTTGGCTGCCACAAGCCAGTTATCCCTGTGGTAACTTTTCT
TATGATAGGAAGAGCCGACATCGAAGGATCAAAAAGCAACGTCGCTATGAA
GTGAACAATCCAACGCTTACCGAATTCTGCTTCGGTATGATAGGAAGAG
TCTAAACCCAGCTCACGTTCCCTATTAGTGGGTGAACAATCCAACGCTTA
GACCTTCATCAGTAGGGTAAAACTAACCTGTCTCACGACGGTCTAAACC
TTCCTCTCGTACTAAATTGAATTACCGTTGCGGCGACCTTCATCAGTAGG
GCCGCAAAAACCAATTATCTGAATCAACGGTTCCTCTCGTACTAAATTG
CAGCCATAATCCGGCAGATGGTAGCGTCGCGGCACTGCCTGGTCAGACAGCCGCAAAAAC
CTTAGAGGCGTTCAGCCATAATCCGGCAGATGGTAGCGTC
CGACGTGCGGGGCGAAATCGCCGCGTTCCGGCACGGATTCTGACTTAGAG
GCCTGCTGAGGTGCATGGCCCGAAGGCCTATTCGTATCCAACTACGACGTGCGG
CAATCCGCTAGCCAGCTCCCGAGGGTTTCTCCCCGGGAGCCGTCGCCAGCCTGCTG
CTAACTCACCGGCGTGTGGCTTGAGTACGGCTGAGCGGAC
AATTTGCAATTCACATTACGTATCGCATTTCGCTGCGTTCTTCATCGATGCC
CGCAATGTGCGTTCAAAGACTCGATGATTCACTGAATTTGCAATTCACAT
CCCCCCGGAATACCAGGGGGCGCAATGTGCGTTCAAAGAC
CAACACACAAGCCGGGCTTGAGGGCAGCAATGACGCTCGGACAGGCATGCC
GCCCGTCCCCCGGAGATCGGAGGACGGGGCCCAACACACAAGCCGGGCTT
GGTGCCGCCGCTGCCTTTCGGGCCCGTCCCCCGGAGATCG
GGCCCGCCCGCGGGGAAATACACCGGCACGAATGCCGGCTGAACCCCGC
ACCGACCGCCCAAACCGACGCTGGCCCGCCCGCGGGGAAA
TAACACGCTTAAGCGCCATCCATTTTCAGGGCTAGTTCATTCGGCCCGTGAGTTGTT
CATCGTTTCTACCCTGACGGCGAGGTATGGGTAACACGCTTAAGCGCCATCCATTT
CTTCGTCACGGACCCCCACGCCTGCCTACTCGTCAGGGCATCGTTTCTACCCTGAC
AAATCACATTGCGTCAACACCACTTTCTGGCCATCGCAATGCTATGTTTTA
CTTGGTTGAATTTCTTCACTTTGACATTCAGAGCACTGGGCAGAAATCAC
TTAAGAGAGTCATAGTTACTCCCGCCGTTTACCCGCGCTTGGTTGAATTTCTT
GCGTCACTAATTAGATGACGAGGCATTTGGCTACCTTAAGAGAGTCATAG
GACAGTGGGAATCTCGTTAATCCATTCATGCGCGTCACTAATTA
CGCTGATTCTGCCAGGCCCGTTCCCCTGGCTGTGGTTTCGCTAGATAGTAGATAGG
AGGGTCTTCTTTCCCCGCTGATTCTGCCAGGCCCGTTCCC
TGGCGCCGAAGCTCCCACCTATTCTACACCCCATATGTCTTTTCACAATGTCAAACTAGA
GTGGTATTTCACTGGCGCCGAAGCTCCCACCTATTCTACA

TGGGCGGTGAAGCCCAGTTCCGCTTCATTGAATAAGTAAAAAAACGATAAAGG
AACTCACCGGCGTGTGGCTTGAGTACGGCTGAGCGGACGG
ACATACAACAGTGAGGATTCGCTGGTGGTCACCCACCCAA
ACAAGTTGCTTATACTTAGACATGCATGGCTTAATCTTTGAGACAAGCA
ACGATAACTGATTTAATGAGCCATTCGCAGTTTCACAGTACAAGTTGCTT
ACCACAGGTATCCATGTAGTAAGGTACTATCAAATAAACGATAACTGA
CGGGGTTTTTAGCATGTATTAGCTCTAGAATTACCACAGGTATCC
TTTTTATCTAATAAATACACCCCTTCCTGAAGTCGGGGTT
GATTCGTTAAGTTATTATGATTCACCAAGGAGCCCCGAAGGGCGTTGGTTT
AAATTTGAATGAACCATCGCCGGCGCAAGGCCATGCGATTCGTTAAGTTATTAT
CATGGTAGGCCACTATCCTACCATCGAAAGTTGATAGGGCAGAAATTTGA
CTAATTCCCCGTTACCCGTTGCCACCATGGTAGGCCACTATCCTA
GATGTGGTAGCCGTTTCTCAGGCTCCCTCTCCGGAATCGAACCCTAATTC
GATTGGGTAATTTGCGCGCCTGCTGCCTTCCTTGGATGTG
GAGCCCCGTATCAGTATTTATTGTCACTACCTCCCCGTAT
TGTTCCTCGTTAAGGGATTTAAATTGTTCTCATTCCAATTACGAGACCCA
CGGACCGGCCAGCCAGACCCAAGGTTCAACTACGAGCTTTTTAACTGCAACA
CCCAGAAGGAAAGGTCCAGCCGGACCAGTACTCGCGGTGAGGCGGACCGG
CCAGTGAAGGCCATGAGGTTCCCCAGAAGGAAAGGTCCAG
TCAATTTCTTTAAGTTTCAGCCTTGCGACCATACTCCCCCCAGAACCCAAAAA
CCTTGTGGTGCCCTTCCGTCAATTTCTTTAAGTTTCAGCC
GTGTTGAGTCAAATTAAGCCGCAGGCTCCACGCCTTGTGG
AATCTGTCAATCCTTATTTTGTCTGGACCTGGTGAGTTTCCCCGTGTTGA
GCACCACCATCCAAAAGATCAAGAAAGAGCTCTCAATCTGTCAATCCTTA
AATTAAGCAGACAAATCACTCCACCAACTAAGAACGGCCATGCACCACCAT
TATTTAAGGGCCGAGGTCTCGTTCGTTATCGCAATTAAGCAGACAAATCA
CCTAAGAAGCCAGCGGCCCGCAAATGCGGACCGGGCTATTTAAGGGCCGAGGTC
GCTTGAGCCGATAGTCCCCCTAAGAAGCCAGCGGCCCGCA
CAGAACATCTAAGGGCATCACAGACCTGTTATTGCCGCGCACTTCCATC
TGATGTACTCGCTGGCCCTGTCAGTGTAGCGCGCGTGCGGCCCAGAACATCTAAGGGCAT
ACAAGATTACCCAAACCTCTCGGTTAAGGTGATGTACTCGCTGGCCCTGTCAGTG
AGCACGACAGGGTTTAACAAGATTACCCAAACCTCTCGGT
GTGCCTACTAGGCATTCCTCGTTGAAGAGCAATAATTGCAATGCTCTATC
GTAATCGGCACGAGCTGATGACTCGTGCCTACTAGGCATTC
TGAGCCATTCAATCGGTAGTAGCGACGGGCGGTGTGTACAAAGGGCAGGGACGTAAT
TTGCCAACCCTCCTAAGCCAATCCCAAGGCCTCACTGAGCCATTCAATCGGTAGTAGC
CGAGTTTGACCAAGTTTTCGGCTCTGGGGGGTCGTTGCCAACCCTCCTAAGC
TTTACTTCCTCTAAATGACCGAGTTTGACCAAGTTTTCGG
AATGATCCTTCCGCAGGTTCACCTACGGAAACCTTGTTACGACTTTTACT
TGGCGCCGGAGCTCCCACCTATTCTACACCCCATATGTCTTTTCACAATGTCAAACTAGA
GTGGTATTTCACTGGCGCCGGAGCTCCCACCTATTCTACA
GTGAAGCCCAGTTCCGCTTCATTGAATAAGTAAAAAAACGATAAAGGTAGTGG
AAGGACCTTAACGCTAGAAGATGGGCGGTGAAGCCCAGTTCCGCTTCA
TCTTCAACCCGGATCGGCCCGCGAAGGACCTTAACGCTAG
CCCAGAAGGAAAGGCCCAGCCGGACCAGTACTCGCGGTGAGGCGGACCGG

CCAGTGAAGGCCATGAGGTTCCCCAGAAGGAAAGGCCCAG
TGCTTTGAACACTCTAATTTTTCACAGTAAAAGTCCTGGTTCCCCCCACAGCC
TCCATGCTAATGTATTCGAGCAAAGGCCTGCTTTGAACACTCTAATTTTT
AACAAAATAGAACCACACGTCCTATTCTATTATTCCATGCTAATGTATTCG
TTACGGCGGTCCTAGAAACCAACAAAATAGAACCACACGT
AAATCCAAGAATTTCACCTCTGACAGCTGAATACTGACGCCCCCGACTATCCCTATT
AGTTAGTCTTCAGCAAATCCAAGAATTTCACCTCTGACAG
TTCCCTGATTAATGAAAACATCCTTGGCGAATGCTTTCGCAGTAGTTAGT
CGGTATCTGATCGTCTTCGATCCCCTAACTTTCGTTCCCTGATTAATGAA
CCTAGTCGGCATAGTTTATGGTTAAGACTACGACGGTATCTGATCGTCTT
GCCGAACGGGTCATTATAGAATCCCGTCCGATCCCTAGTCGGCATAGTTT
GTTCTTCATCGATGCCGGAACCAAGAGATCCGTTGTTGAAAG
TCGATGATTCACTGAATTTGCAATTCACATTACGTATCGCATTTCGCTGC
GGGGGCGCAATGTGCGTTCAAAGACTCGATGATTCACTGA
CAAGCCGTGCTTGAGGGCAGCAATGACGCTCGGACAGGCATGCCCCCCGGAATACCAGGG
GGGCCCAACACACAAGCCGTGCTTGAGGGCAGCAATGACG
TTTCGGGCCCGTCCCCCGGAATCGGAGGACGGGGCCCAAC
CATACGCTCGAGGACCGGACGCGGTGCCGCCGCTGCCTTTC
TAAAAATTTGGGTTGATCGGCAAGCGCCGGCCGGGCCTACAGAGCGGGTGACAAA
GGGTATCCCTACCTGATCCGAGGTCAACCTGGATAAAAATTTG
TTGGTTTCTTTTCCTCCGCTTATTGATATGCTTAAGTTCAGCGGGTATCC
TTTCAAATTTGAGCTCTTGCCGCTTCACTCGCCGTTACTGGGGCAATCCCTGTTGGT
CCCGAAGGAGCCAGCTTTCAAATTTGAGCTCTTGCCGCTT
GACCGCTCCCGAAGCATCCTCTGCAAATTACAATGCGGAC
GATTCTCACCCTCTATGACGTCCCGTTCCAGGGCACTTAGATG
TCGACTCGTCGAAGGAGCTTCACACGGGCGCGGACACCCCATCCCATACGGGATTCTCA
CGCGGGGAAGTACACCGGCACGAATGCCGGCTGAACCCCGCGAGCGAGTCTGGTCGC
CGACGCTGGCCCGCCCGCGGGGAAGTACACCGGCACGAAT
GAACCGCTACGAGCCTCCACCAGAGTTTCCTCTGGCTTCGCCCTATTCAGGCATAGTTCA
ATTTGCACGTCAGAACCGCTACGAGCCTCCACCAGAGTTT
TACCAGATGGTTCGATTAGTCTTTCGCCCCTATACCCAAATTCGACGATCGATTTG
TTCGGCAGGAACCAGCTACCAGATGGTTCGATTAGTCTTT
AATTCGCGTTACTGCTATCCTGAGGGAAACTTCGGCAGGA
CAACCCCAAGGCCTCTAATCATTCGCTTTACCTCATAAAA
TTAAGCAACAAGGGCTTCTTACATATTTAAAGTTTGAGAATAGGTTAAGGTT
ATTCTAATGCCCACGTTCAATTAAGCAACAAGGGCTTCTT
CCAGTTCTGCTTACCAAAAATGGCCCACTAGTAACGCATCATTCTAATGC
TTTTGTGGTGTCTGATGAGCGTGTATTCCGGCACCTTAACCTCGCGTTCGGTTCAT
GTCGGGCTGTCTAGATGAACTAACACCTTTTGTGGTGTCTG
TGAGTTGTTACACACTCCTTAGCGGATTCCGACTTCCATGGCCACCGTCGGGCTGT
CTAGTTCATTCGGCCCGTGAGTTGTTACACACTCCTTAGCGG
TGGGTAACACGCTTAAGCGCCATCCATTTTCAGGGCTAGTTCATT
CCTGCCTACTCGTCAGGGCATCGTTTCTACCCTGACGGCGAGGTATGGGTAA
AAGATCTGCACTAGGGGCCGTTCGACCCGGGATCACTCCCAAGGCTTCGT
TGAGTATTTGCTACTACCACCAAGATCTGCACTAGGGGCC

ACCTTTCCCCACTTCAGTCCTCAAAGTTCTCATTTGAGTATTT
CTATGCCTTAGGATCGACTAACCCATGTCCAACTGCTGTTCACATGGAACCTTTCCCCA
GAGGGCGCCTTTAAAACGGAACTTCCCTATGCCTTAGGATCGACTAACCCATG
CTTCCCTTTCGGCAAACGGCGCACGAGGGCGCCTTTAAAACGGAA
ATCTAGGTGCCGGAATGTTAACCGGCTTCCCTTTCGGCAA
AGGACCCCCGCCGACGTCTCCGCGTTCAGTTACGTTGCCGTGGAGAATCC
GAAGAAAAGAGAACTCTTCCCAGGACCCCCGCCGACGTCT
CAAACCGATTTCAGGGTGATAGGCTGTCAAGAAGAAAAGA
GGGCGCACCGGACGCCGCAAAAGTGCGGCGCTCTGCCAGCCATAGAACCCTAGCTCCGG
AAGGGTCGTCGGGGGCGCACCGGACGCCGCAAAAGTGCGG
GCGTGAAAACTATTCCTTCCCGCGGATTTTCAAGGGTCGT
TTCTATCAACTAGAGGCTGTTCACCTTGGAGACCTGCTGCGGTTATGAGTACGA
TTTGCCGACTTCCCTTATCTACATTGTTCTATCAACTAGAGGCTGTTCA
CTTAGAGCCAATCCTTATCCCGAAGTTACGGATCCATTTTGCCGACTTC
CTGCTCCAGGAGGTTCCCCCCAAGGCCCAGCGAGCCCGACCCTTAGAGCCAATCC
CAAGGGCGTCCCCAACCCGGTGCTGCGGGGCGCCGGCCGG
CTAAGTTGGTCGTTAATCGCCCGCCGGACGGCCGAAGCCTGCCAAGGGCGT
TCCCCTTGTCCGTACCAGTTCTAAGTTGGTCGTTAATCGC
CACCACTTTCTGGCCATCGCAATGCTATGTTTTAATTAGACAGTCAGATT
TTTGACATTCAGAGCACTGGGCAGAAATCACATTGCGTCAACACCACTTTCT
GTTTACCCGCGCTTGGTTGAATTTCTTCACTTTGACATTCAGAGCA
GACGAGGCATTTGGCTACCTTAAGAGAGTCATAGTTACTCCCGCCGTTTA
AATCTCGTTAATCCATTCATGCGCGTCACTAATTAGATGACGAGGCATTT
GCCCGTTCCCCTGGCTGTGGTTTCGCTAGATAGTAGATAGGGACAGTGGGAATCTCG
GTCTTTTCACAATGTCAAACTAGAGTCAAGCTCAACAGGGTCTTCTTTCC
TTTCACTGGCGCCGAAGCTCCCACCTATTCTACACCCCATATGTCTTTTC
AAAACGATAAAGGTAGTGGTATTTCACTGGCGCCGAAGCT
GACCTTAACGCCAGAAGATGGGCGGTGAAGCCCAGTTCCGCTTCATTGAATAAGTAA
CAGCCAAACTCCCCACCTGACAATGTCTTCAACCCGGATC
CATGAGTCCCCCTTAGGACACCTGCGTTGTTGTTTAACAGATGTGCCGCCCCAGCCAA
CCTTTTGTTCTACTGGAGATTTCTGTTCTCCATGAGTCCCCCTTAGGACACCTGCG
TCAAGGGGACTTTTACCCTTTTGTTCTACTGGAGATTTCTG
AGGCCACACTTTCATGGTTTGTATTCACACTGAAAATCAAAATCAAGGGG
CTAGCCTCAAATTTCGAGGGACTAAAGGATCGATAGGCCACACTTTCATG
CAGTTATCCCTGTGGTAACTTTTCTGGCACCTCTAGCCTCAAATTTCGAG
ATCAAAAAGCAACGTCGCTATGAACGCTTGGCTGCCACAAGCCAGTTATC
GAATTCTGCTTCGGTATGATAGGAAGAGCCGACATCGAAGGATCAAAA
CTATTAGTGGGTGAACAATCCAACGCTTACCGAATTCTGCTTCG
TAAAACTAACCTGTCTCACGACGGTCTAAACCCAGCTCACGTTCCCTATTA
AAATTGAATTACCGTTGCGGCGACCTTCATCAGTAGGGTAAAACTAACCTGTC
TTATCTGAATCAACGGTTCCTCTCGTACTAAATTGAATTACCGTTGCGGC
CACTGCCTGGTCAGACAGCCGCAAAAACCAATTATCTGAATCAACGGTTC
AGGCGTTCAGCCATAATCCGGCAGATGGTAGCGTCGCGGCACTGCCTGGTC
CGGCACGGATTCTGACTTAGAGGCGTTCAGCCATAATCCG
CAACTACGACGTGCGGGGCGAAATCGCCGCGTTCCGGCAC
